# Epigenome-wide DNA methylation profiling of conditioned pain modulation in individuals with non-specific chronic low back pain

**DOI:** 10.1186/s13148-022-01265-z

**Published:** 2022-03-26

**Authors:** Burel R. Goodin, Demario S. Overstreet, Terence M. Penn, Rahm Bakshi, Tammie L. Quinn, Andrew Sims, Travis Ptacek, Pamela Jackson, D. Leann Long, Edwin N. Aroke

**Affiliations:** 1 Department of Psychology, College of Arts and Sciences, University of Alabama at Birmingham, Birmingham, AL USA; 2 Center for Addiction and Pain Prevention and Intervention (CAPPI), University of Alabama at Birmingham, Birmingham, AL USA; 3 Department of Biostatistics, School of Public Health, University of Alabama at Birmingham, Birmingham, AL USA; 4 Center for Clinical and Translational Science, University of Alabama at Birmingham, Birmingham, AL USA; 5 Department of Acute, Chronic and Continuing Care, School of Nursing, University of Alabama at Birmingham, 1701 University Boulevard, Birmingham, AL 35294 USA

**Keywords:** Conditioned pain modulation, Nonspecific chronic low back pain, Central sensitization, Reduced representation bisulfite sequencing, DNA methylation, Epigenetics, RRBS

## Abstract

**Background:**

The pathoanatomic cause of chronic low back pain (cLBP) cannot be identified for up to 90% of individuals. However, dysfunctional processing of endogenous nociceptive input, measured as conditioned pain modulation (CPM), has been associated with cLBP and may involve changes in neuronal gene expression. Epigenetic-induced changes such as DNA methylation (DNAm) have been associated with cLBP.

**Methods:**

In the present study, the relationship between CPM and DNAm changes in a sample of community-dwelling adults with nonspecific cLBP (*n* = 48) and pain-free controls (PFC; *n* = 50) was examined using reduced representation bisulfite sequencing. Gene ontology (GO) term enrichment and Kyoto Encyclopedia of Genes and Genomes (KEGG) pathway analysis were applied to identify key pathways involved in efficient versus deficient CPM.

**Results:**

Based on CPM efficiency, we identified 6006 and 18,305 differentially methylated CpG sites (DMCs) with *q* values < 0.01 among individuals with cLBP and PFCs, respectively. Most of the DMCs were hypomethylated and annotated to genes of relevance to pain, including *OPRM1*, *ADRB2*, *CACNA2D3*, *GNA12*, *LPL*, *NAXD*, and *ASPHD1*. In both cLBP and PFC groups, the DMCs annotated genes enriched many GO terms relevant to pain processing, including transcription regulation by RNA polymerase II, nervous system development, generation of neurons, neuron differentiation, and neurogenesis. Both groups also enriched the pathways involved in Rap1-signaling, cancer, and dopaminergic neurogenesis. However, MAPK-Ras signaling pathways were enriched in the cLBP, not the PFC group.

**Conclusions:**

This is the first study to investigate the genome-scale DNA methylation profiles of CPM phenotype in adults with cLBP and PFCs. Based on CPM efficiency, fewer DMC enrichment pathways were unique to the cLBP than the PFCs group. Our results suggest that epigenetically induced modification of neuronal development/differentiation pathways may affect CPM efficiency, suggesting novel potential therapeutic targets for central sensitization. However, CPM efficiency and the experience of nonspecific cLBP may be independent. Further mechanistic studies are required to confirm the relationship between CPM, central sensitization, and nonspecific cLBP.

**Supplementary Information:**

The online version contains supplementary material available at 10.1186/s13148-022-01265-z.

## Introduction

For years, low back pain has been one of the most common health problems and a leading cause of years lived with disability worldwide [1]. It is estimated that about 70 to 85% of adults would experience low back pain at some point in their lives, with a global point prevalence of about 7 percent [2, 3]. For many individuals, low back pain lasts more than 12 weeks, progressing to chronic low back pain (cLBP). Unfortunately, the pathoanatomic cause of cLBP cannot be identified for up to 90% of these individuals [2, 4, 5]. Nonspecific cLBP pain is associated with major depression, decreased productivity, disability, and reduced life quality [6, 7]. Therefore, there is an urgent need for research to better understand the underlying mechanisms that cause and sustain nonspecific cLBP and guide effective treatments.

Contemporary evidence suggests that alterations in the endogenous processing of nociceptive input may play an essential role in cLBP [5, 8, 9]. In humans, the descending endogenous inhibitory system can inhibit nociceptive impulses via second-order afferent neurons in the dorsal horn [10]. These pain modulatory mechanisms mediate cognitive processes between the limbic system and higher brain functions in the prefrontal cortex that control nociceptive signals [5]. The effectiveness of descending endogenous pain modulation is commonly assessed in humans through the “pain inhibits pain” approach known as conditioned pain modulation (CPM) [8, 11].

A growing number of studies have reported dysfunctional (i.e. deficient) CPM in individuals with cLBP [9, 12–15]. Specifically, it has been suggested that enhanced pain sensitivity and deficient CPM correlate with greater cLBP severity and disability [16]. More recently, two systematic reviews found that individuals with cLBP have significantly reduced CPM [17] and pressure pain thresholds (PPTs) [8]. Thus, it is possible that nonspecific cLBP is related to alterations in descending pain modulatory pathways. Other investigators have suggested that CPM testing could uncover hidden features of pain modulation in individuals with and without chronic pain conditions [11]. While the exact biological mechanisms responsible for developing these neurophysiologic changes in nonspecific cLBP are not well understood, long-lasting structural and functional adaptations induced by neuronal activity appear to be dependent on alterations in gene expression [18–20].

Long-term regulation of gene expression associated with chronic pain can be rooted in epigenetic mechanisms such as DNA methylation (DNAm). DNAm is a stable epigenetic modification through which environmental and experiential factors regulate genome function (gene expression) and the associated phenotype (e.g., pain sensitivity) without changing the DNA sequence [21, 22]. Studies have demonstrated that pathological manifestations of chronic pain such as neuronal plasticity, which sustains central sensitization, may be supported by epigenetically induced gene expression changes [18]. Other studies have shown that DNAm changes in pain-related genes play a role in many chronic pain conditions, including fibromyalgia [23], migraines [24], and cLBP [25].

We recently demonstrated that differentially methylated genes enriched pain-related genomic pathways in adults with cLBP compared with pain-free controls (PFCs) [26]. The primary objective of this study was to explore the relationship between CPM and DNAm changes in a sample of community-dwelling adults with nonspecific cLBP. We also determined the functional pathways enriched by genes annotated to differentially methylated CpGs (DMCs) in patients with efficient versus deficient CPM.

## Methods

This study was part of an on-going project investigating racial/ethnic and socioeconomic status differences in cLBP severity and disability: “Examining Racial and SocioEconomic Disparities (ERASED) in cLBP (R01MD01044).” More details concerning ERASED can be found elsewhere [27, 28]. First results of the study concerning differential DNAm and functional enrichment pathways have been published, and this article uses the same patient samples [26]. All participants provided informed consent before their inclusion in the study. The Institutional Review Board (IRB) at the University of Alabama at Birmingham approved the study.

### Study design

Fifty community-dwelling adults ages 18 to 85 years with nonspecific cLBP and 50 pain-free controls (PFCs) participated in this study. Potential participants with cLBP were screened for eligibility via telephone and electronic medical records. Participants were diagnosed with nonspecific cLBP using the joint clinical practice guidelines from the American Colleges of Physicians and the American Pain Society [29]. Included participants were able to read, write, and understand the English Language. Participants self-identified as non-Hispanic Black/African American or non-Hispanic White/Caucasian. The following exclusion criteria were applied: low back pain attributable to infection, trauma, malignancy, or ankylosing spondylitis, systemic infection, chronic inflammatory disease (e.g., rheumatoid arthritis, systemic lupus erythematosus, fibromyalgia), poorly controlled diabetes, neurological disorders (e.g., Parkinson’s, multiple sclerosis, and epilepsy) and severe psychiatric disease requiring hospitalization within the past 12 months. The same inclusion and exclusion criteria applied cLBP and PFC participants, except the diagnosis of cLBP in the PFCs.

### Laboratory-test procedure

Eligible participants (cLBP and PFC) completed two laboratory study sessions approximately seven days apart. During the first study session, participants self-reported sociodemographic information, including age, sex, race, and social status. Additionally, participants provided information about pain medications (prescription and over-the-counter) and underwent quantitative sensory testing (QST). During the second study session, participants provided venous blood for epigenetic analysis and completed the pain questionnaire. Given that half of the participants had a diagnosis of cLBP, we did not ask the participants to withhold their daily pain medications. Withholding the pain medication could have confounded pain perception. Instead, a current list of all pain medications was recorded and included in statistical analysis as needed.

### Brief pain inventory (BPI)—short form

Using the BPI-scale, participants self-reported their perceptions of cLBP severity and interference in daily activities. This instrument includes four items that ask participants to rate their worst, least, average, and current pain in the last 24 h on a 11-point numeric rating scales (0 = no pain and 10 = worst pain imaginable). The average of the four items corresponds to pain severity. Participants provide a rating about their mood, walking ability, work, relationship to other, sleep, and enjoyment of life during the last 24 h. The average of these items was used to determine pain interference. For our study sample, BPI had an internal consistency Cronbach’s alpha of 0.93.

### Quantitative sensory testing

#### Pressure pain threshold (PPT)

To determine the participant’s baseline pressure pain threshold (PPT), a handheld algometer (Medoc, Ltd., AlgoMed, Ramat Yishai, Israel) was applied to the erector spinae muscle at the lower back, and the pressure was gradually increased at a rate of 30 kilopascals per second (kPa/s). Participants indicated when the increasing pressure stimulation first became painful, and PPTs were measured in kilopascals (kPa). This procedure was repeated three times, and the mean of the three readings calculated as the baseline PPT.

#### Test of CPM

After determining the baseline PPT, CPM was assessed using the sequential heterotopic noxious conditioning stimulation paradigm with algometry as the test stimulus and noxious cold water as the conditioning stimulus [30]. Each participant was instructed to immerse the non-dominant hand, up to the wrist, in a bath of water cooled by a refrigeration unit (ARTIC A25, ThermoFisher Scientific, USA) that maintained a water temperature at 12 °C for 60 s. Participants were told they could remove their hand from the water if the pain became unbearable. None of the participants in this study removed their hands before the allocated 60 s duration. After 60 s of immersion and following removal of the hand from the cold water, the algometer was again applied to the erector spinae muscle at the lower back. Participants again indicated when the increasing pressure stimulation became painful. This procedure was repeated twice with a two-minute rest period between each test on each participant and the average pressure calculated (conditioned PPT). A CPM score was calculated using the following formula:

$$\%\, {\text{Change}}\,\,{\text{CPM}} = \left( {\frac{{{\text{Conditioned}}\,\,{\text{PPT}} - {\text{Baseline}}\,\,{\text{PPT}}}}{{{\text{Baseline}}\,\,{\text{PPT}}}}} \right) \times 100$$

For this study, a positive percent change in CPM score designates an efficient pain modulation (i.e., the presence of pain inhibition) or a reduction in the painfulness of the test stimulus by the conditioning stimulus. In contrast, a negative percent change in CPM or a score of 0%change indicates a deficient pain modulation (i.e., the presence of pain facilitation or lack of inhibition).

### DNA isolation and DNA methylation sequencing

As previously reported, the Heflin Center Genomics Core at the University of Alabama performed genomic DNA extraction from buffy coat samples and RRBS [26]. Briefly, genomic DNA extraction was performed using the *Gentra Puregene* DNA Purification Protocol (Qiagen, Valencia, CA, USA). DNA quantity and quality were assessed using a Qubit fluorometer and Bioanalyzer (Agilent Technologies, Santa Clara, CA), respectively. RRBS libraries were prepared using the Ovation RRBS Methyl-Seq kit (Tecan Genomics, Redwood City, CA, USA), which uses Qiagen Epitect bisulfite conversion Kit (Qiagen, Valencia, CA, USA) on the Illumina NextSeq 500 platform. Raw reads of the DNAm profiles were generated according to standard Illumina protocols.

### Data processing and analysis

Demographic variables were compared between efficient and deficient CPM groups using Mann–Whitney *U* test for continuous variables and Pearson’s Chi-Square test for categorical variables.

DNAm pre-processing was performed as previously described [26]. Briefly, quality control of the RRBS data was performed using FastQC (version 0.11.5, Babraham Bioinformatics, UK). Raw RRBS reads were cleaned, and adapters removed using Trim Galore [31]. Bismark was used to align and map the trimmed forward reads to the human reference genome (hg38) [32]. The reverse reads contained only the unique molecular identifier sequence. Differential methylation analysis at the nucleotide level was performed, and summary statistics of read depth were obtained using methylKit 1.10.0 [33] in R 3.6.2. Differential methylation was tested between conditioned pain modulation phenotypes for adults with cLBP and PFCs. We controlled for age, sex, self-identified race using native functionality in methylKit. DMCs with FDR corrected *p*-values (*q*-values) less than 0.01 were considered significant. All DMCs were annotated with gene symbols using genomation (version 1.16.0) [34] in R 3.6.2. Annotations for hg38 of genes and CpG islands were obtained from the UCSC genome browser (genome.ucsc.edu) [35]. For genomic-wide methylation analysis, differentially methylated loci (DML) were defined as DMCs with *q*-values < 0.01 and methylation differences of at least 10 percent.

Genes associated with significant DMCs were used for functional enrichment analyses to identify gene ontologies (GO) and functional pathways overrepresented genes. For each comparison, genes associated with significant DMCs were used for functional enrichment analyses to determine the overrepresented GO and functional pathways in genes associated with the DMCs. We further divided the DMCs into hypomethylated and hypermethylated gene lists and determined the overrepresented GO and functional pathways in genes associated with hypomethylated or hypermethylated DMCs. The gene lists were analyzed using gprofiler2 (version 0.2.0) [36] in R 3.6.2. After controlling for multiple testing, associations with adjusted *p*-value less than 0.05 were considered significant.

## Results

Two participants in the cLBP group selected multiple racial categories and were excluded from the analysis. The final sample included forty-eight adults with cLBP (mean age 44.52 ± 12.95 years, 56.3% females) and 50 adult PFCs (mean age 39.66 ± 14.51 years, 48% females). The mean age of the 98 participants included in this study was 42.04 (SD = 13.91) years. The average baseline PPT was 510.02 ± 233.1 kPa/s. The change in CPM ranged from − 48.52 to 0 percent for deficient CPM group, and 0.86 to 91.43 percent for efficient CPM group. No significant difference between gender (Chi-square = 0.30, *p* = 0.58), age (*U* = 1146.50, *p* = 0.874), race (Chi-square = 0.07, *p* = 0.79) or cLBP status (Chi-square = 0.001, *p* = 0.97) were found between efficient and deficient CPM groups.

Given that other investigators have previously associated CPM with cLBP, we separated cLBP from PFCs in subsequently analysis. Among participants with cLBP, those with deficient CPM showed a significant decrease PPT (mean = − 15.75, SD = 12.71) during the cold test when compared to the efficient group which showed an increase in PPT (mean = 30.48, SD = 22.51). This group difference was statistically significant (*p* < 0.001). Similarly, among PFCs, participants in the deficient group showed a significant decrease in PPT (mean = − 14.96, SD = 12.73) compared to the efficient group which showed a significant increase in PPT (mean = 17.51, SD = 14.63; *p* < 0.001). As summarized in Table 1, there were no significant differences between the efficient and deficient groups in terms of sex, age, race, pain severity, and pain interference (*p* > 0.05).

**Table 1:** Baseline demographic data

	Deficient CPM (*n* = 41)	Efficient CPM (*n* = 57)	*p*-value
Pain status			
cLBP	20 (48.8)	30 (50.8)	0.84^$^
PFCs	21 (51.2)	29 (49.2)	

Chronic low back pain	*N* = 20	*N* = 28	
Mean age (SD, years)	44.50 (14.21)	44.54 (12.25)	0.93*
Sex (*n*, %)			
Male	9 (45)	12 (42.9)	0.88^$^
Female	11 (55)	16 (57.1)	
Race (*n*, %)			
Black	9 (45)	13 (46.4)	0.92^$^
White	11 (55)	15 (53.6)	
Mean CPM (SD)	− 15.75 (12.71)	31.11 (23.02)	< 0.001*
Mean pain severity (SD)	4.20 (1.98)	4.66 (2.61)	0.56*
Mean pain interference (SD)	3.31 (2.33)	3.23 (2.57)	0.88*

Pain free controls	*N* = 21	*N* = 29	
Age (Mean, SD)	41.80 (16.14)	38.61 (13.64)	0.46*
Sex (*n*, %)			0.54^$^
Male	12 (57.1)	14 (48.3)	
Female	9 (42.9)	15 (51.7)	
Race (*n*, %)			0.77^$^
Black	10 (47.6)	15 (51.7)	
White	11 (52.4)	14 (48.3)	
Mean CPM (SD)	− 14.96 (12.73)	17.51 (14.63)	< 0.001*
Mean pain severity (SD)	–	–	
Mean pain interference (SD)	–	–	

cLBP, chronic low back pain; PFC, pain free control; CPM, conditioned pain modulation; SD, standard deviation

^*^Mann–Whitney *U* Test; ^$^Pearson’s Chi-Square Test

### Differentially methylated CpGs based on CPM phenotype in cLBP adults

Through the RRBS analysis, we tested for differences in the distribution of methylation and unmethylation for approximately 6 million CpGs comparing efficient versus deficient CPM groups. We controlled for age, sex, and race of the participant. After correcting for multiple testing, we identified 6006 DMCs sites with q values less than 0.01. Among the 6006 CpG sites, we identified 729 DMLs with at least a 10 percent methylation difference, and *q* < 0.01: 558 were hypomethylated and 171 hypermethylated in individuals with deficient CPM compared to those with efficient CPM. Table 2 shows the top 10 hypo- and hypermethylated loci; many of them are annotated to genes with regulatory functions and relevance to pain pathogenesis.

**Table 2:** Top 10 differentially hypo- and hypermethylated CpGs associated with CPM efficiency in adults with cLBP

Chr	Position	*p*-value	*q*-value	Methylation difference	Genes
Hypomethylated CpGs					
1	7062216	1.99 × 10^–42^	1.84 × 10^–37^	22.97	LOC105376692
1	208071630	1.79 × 10^–42^	1.84 × 10^–37^	29.33	LOC105372887
22	20636231	1.97 × 10^–32^	1.04 × 10^–27^	20.64	POM121L4P
14	101202752	4.65 × 10^–30^	1.72 × 10^–25^	13.84	LINC02285
8	49401429	7.47 × 10^–27^	1.63 × 10^–22^	16.29	LOC100507464
6	4892147	1.66 × 10^–25^	2.87 × 10^–21^	11.90	CDYL
7	152500728	1.71 × 10^–25^	2.87 × 10^–21^	22.73	LOC105375574
20	4873285	7.88 × 10^–25^	1.27 × 10^–20^	24.27	RASSF2
19	47358835	1.35 × 10^–24^	2.08 × 10^–20^	14.80	DHX34
5	170149514	1.98 × 10^–23^	2.72 × 10^–19^	13.26	FOXI1
Hyper methylated CpGs					
12	121444044	2.41 × 10^–45^	8.94 × 10^–40^	− 35.22	MIR7107
5	101611746	7.01 × 10^–43^	1.30 × 10^–37^	− 23.10	LOC105379102
4	183768629	1.09 × 10^–35^	8.09 × 10^–31^	− 23.97	STOX2
3	88191286	6.66 × 10^–33^	4.11 × 10^–28^	− 24.67	C3orf38
14	89052404	1.85 × 10^–30^	8.56 × 10^–26^	− 16.27	LOC105370615
11	7519699	3.60 × 10^–30^	1.48 × 10^–25^	− 17.93	PPFIBP2
2	129828813	1.25 × 10^–29^	4.21 × 10^–25^	− 17.29	LOC107985945
9	30199307	6.57 × 10^–28^	2.03 × 10^–23^	− 23.93	LINC01242
22	21680479	2.33 × 10^–27^	6.65 × 10^–23^	− 19.36	PPIL2
8	19974465	3.11 × 10^–27^	7.69 × 10^–23^	− 16.39	LPL

Differential methylation based on comparison of efficient versus deficient CPM in adults with chronic low back pain

**Fig. 1:**
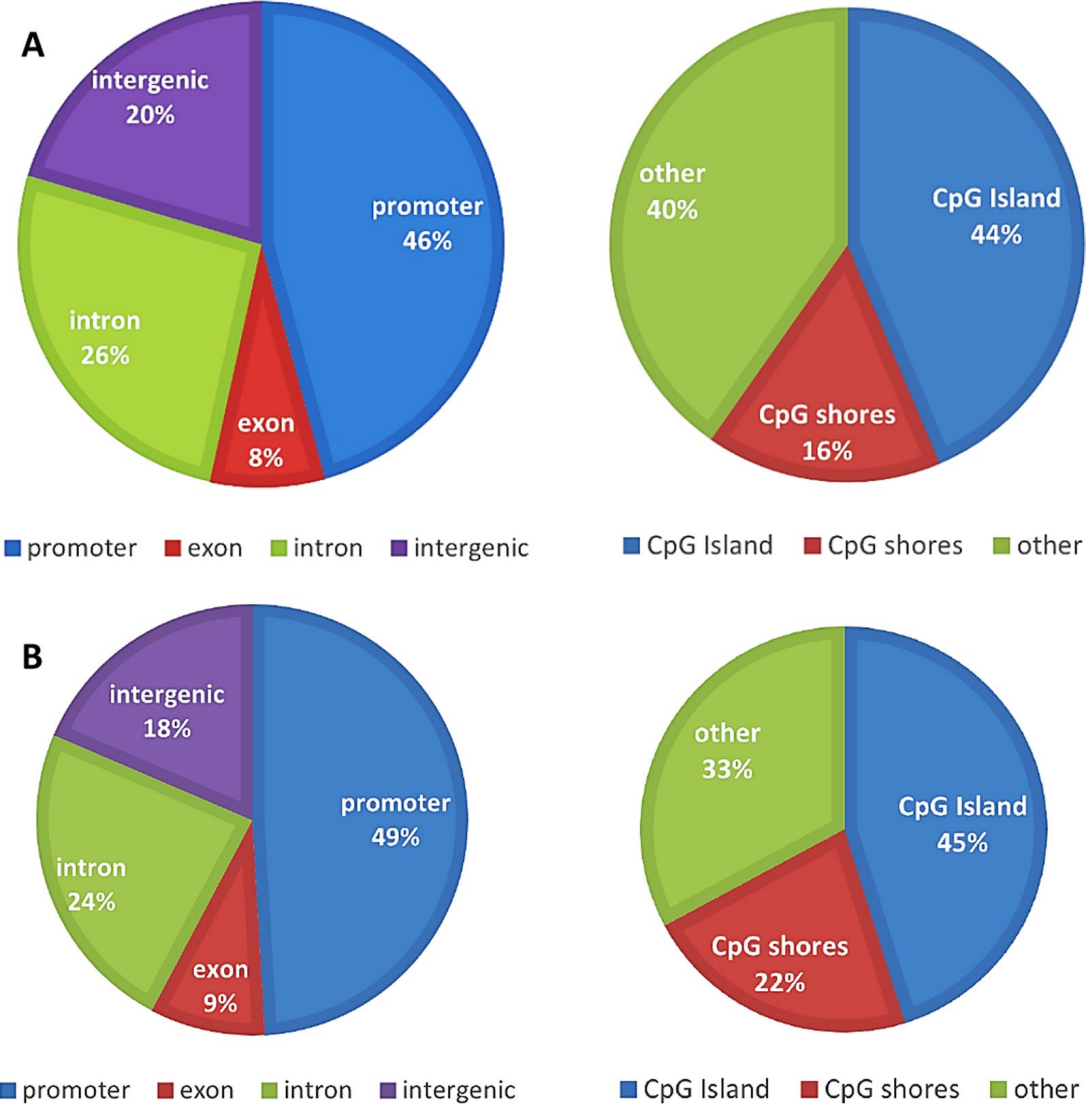
Pie charts showing proportional genomic locations of differential methylated CpGs (DMCs) between individuals with efficient and deficient CPM among participants with cLBP (**A**) and pain-free controls (**B**). The DMCs were mapped to human genome (hg38)

Chr, chromosome; DML, differentially methylated loci;

The DMCs were evenly distributed across all autosomes. As depicted in Fig. 1a, the majority of the DMCs were in promoter (45.77%) or intronic (26.16%) regions. Also, most of the identified DMCs were in CpG island (43.66%) or CpG shores (16.06%). The 13.202 DMCs were annotated to or near to 4671 genes, and these genes were used for significant enrichment.

### Gene ontology enrichment analysis based on CPM phenotype in cLBP adults

**Fig. 2:**
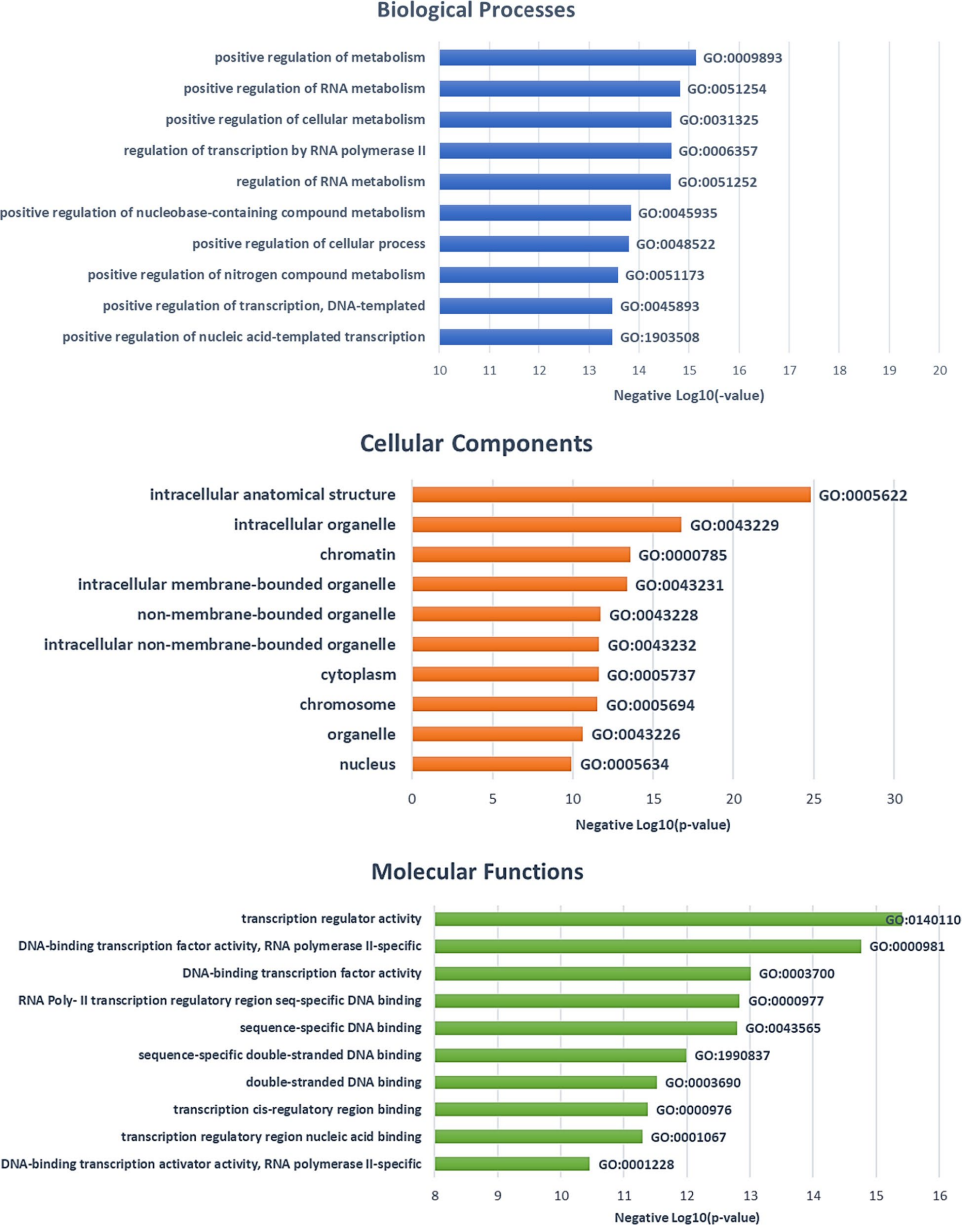
Top 10 GO term enrichment results of DMCs annotated genes between efficient and deficient conditioned pain modulation participants with cLBP. Notes: All depicted GO terms were statistically significant (*p* < 0.05)

After removing duplicates, the DMC annotated genes were entered into a GO enrichment analysis to gain functional insights identifying the most relevant biological processes, molecular functions, and cellular components. After correcting for multiple testing, the DMCs (based on efficient versus deficient CPM) annotated genes significantly enriched 323 GO terms: 251 biological processes, 41 cellular components, and 31 molecular functions (*p* < 0.05). The top 10 GO terms categorized into biological processes, molecular functions, and cellular components are depicted in Fig. 2. Some of the key GO terms with high enrichment scores included transcription regulator activity (GO: 0140110; adj *p* = 3.85 × 10^–16^), positive regulation of metabolic process (GO: 0009893; adj *p* = 7.28 × 10^–16^), DNA-binding transcription factor activity, RNA polymerase II-specific (GO: 0000981; adj *p* = 1.73 × 10^–15)^ and intracellular anatomical structures (GO: 0005622; adj *p* = 1.59 × 10^–25^). Of note, we also identified enrichment of epigenetic regulators such as DNA binding transcription factors and pathways known to play important roles in neuronal differentiation and sensitization (e.g., neuron differentiation, GO: 0030182; adj. *p* = 6.70 × 10^–12^) Additional file 1: Figures S1 and S3 depict GO terms enrichment analysis for genes annotated by hypo- and hyper-methylated CpGs in adults within cLBP.

### Functional pathway enrichment analysis based on CPM phenotype in cLBP adults

**Fig. 3:**
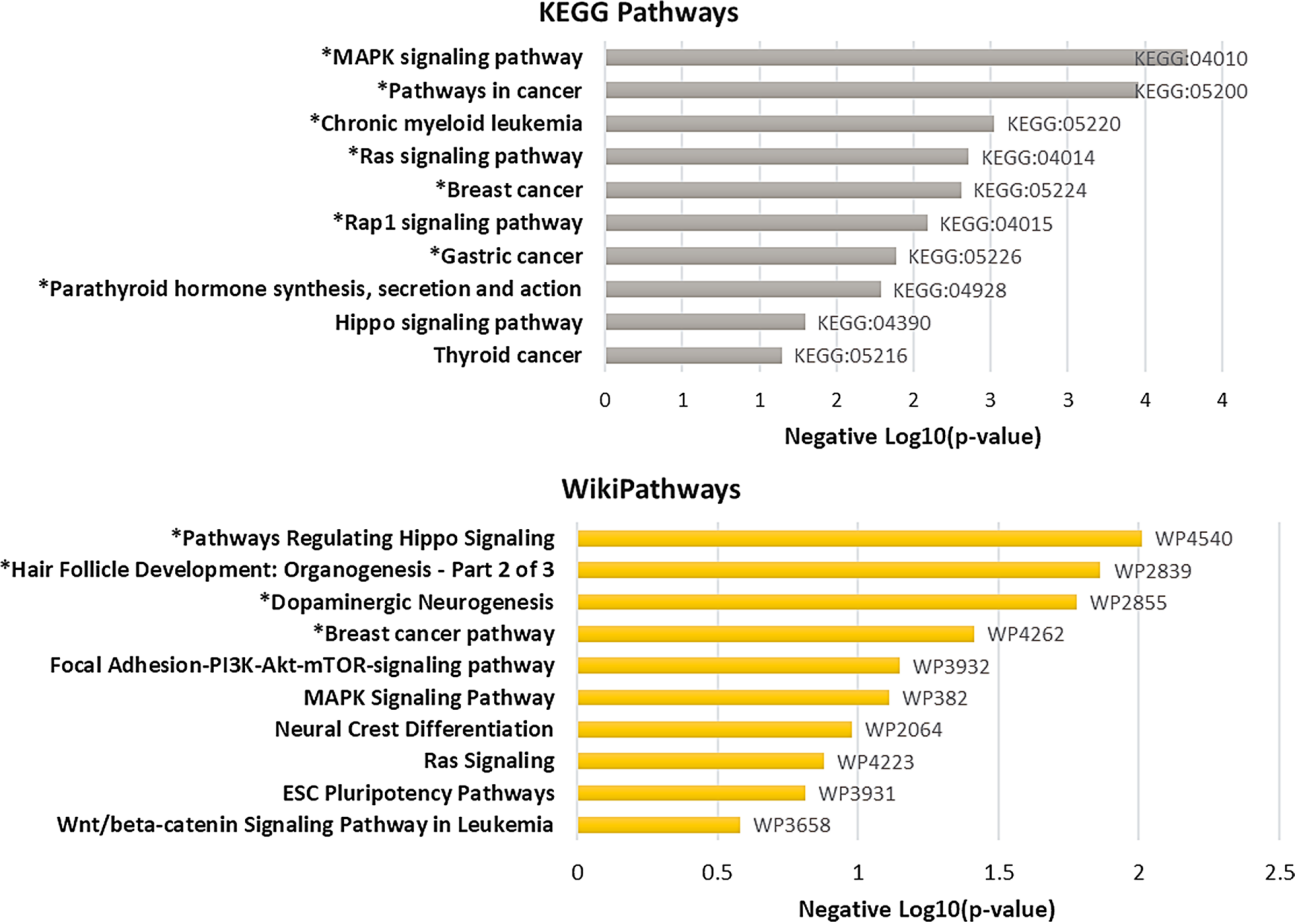
Top 10 KEGG and WikiPathways enrichment results of annotated genes with DMCs between efficient and deficient conditioned pain modulation participants with cLBP. Note: * depicts statistically significant pathways (*p* < 0.05)

In adults with cLBP, functional pathway analysis revealed 12 pathways (8 from KEGG and 4 from wikipathways) significantly enriched by DMCs annotated gene in those with efficient CPM compared with deficient CPM (*p* < 0.05). The top enriched pathways include MAPK signaling pathway (KEGG: 04010; adj *p* = 1.67 × 10^–4^), Ras signaling pathway (KEGG: 04014; adj *p* = 0.004), pathways regulating Hippo signaling (WP4540; adj *p* = 0.01), parathyroid hormone synthesis, secretion and action (KEGG: 04928; adj. *p* = 0.02), and dopamine neurogenesis (WP2855; *p* = 0.02) (Fig. 3). Top ten pathway enrichment analyses for hypo- and hyper-methylated CpGs annotated genes in adults with cLBP depicted in Additional file 1:Figures S2 and S4, and enriched genes are provided in Additional file 2: Tables S1 and S2.

### Differentially methylated loci based on CPM phenotype in PFCs

For comparison, we repeated the differential methylation analysis of efficient versus deficient CPM in PFC adults, controlling for age, sex, and race. After controlling for multiple testing, we identified 18,305 DMCs with *q*-value less than 0.01. Of these DMCs, we identified 305 DMLs in individuals with efficient CPM compared with deficient CPM at q < 0.01 and methylation difference > 10%. Among these, 155 loci were hypomethylated and 150 sites were hypermethylated in individuals with deficient CPM compared with efficient CPM. The top 10 hypo- and hyper-methylated DMCs (sorted by *p*-values) and annotated genes are shown in Table 3. Many genes annotated to these DMLs have previously been shown to be of relevance to pain pathology, including *EEPD1* [37], *MATN1* [38], *EPN3* [39], *CXCL11* [40], and *ASAP2* [41].

**Table 3:** Top 10 differentially hypo- and hypermethylated CpGs associated with CPM efficiency in pain-free control adults

Chr	Position	*p*-value	*q*-value	Methylation difference	Genes
Hypomethylated CpGs					
7	36183317	3.96E−107	7.48E−102	− 30.15	EEPD1
17	50526142	1.21E−89	1.14E−84	− 27.10	EPN3
9	37944491	4.64E−63	2.92E−58	− 30.28	SLC25A51
1	30729051	1.19E−60	5.64E−56	− 31.59	MATN1
17	82551092	6.69E−56	2.11E−51	− 25.58	FOXK2
2	4964659	2.73E−50	5.74E−46	− 14.72	LINC01249
3	13406593	1.70E−49	2.92E−45	− 18.68	NUP210
2	148201344	8.69E−49	1.26E−44	− 15.33	LOC105373673
13	68877143	1.14E−48	1.53E−44	− 21.58	LINC02342
11	23941356	3.49E−48	4.40E−44	− 22.50	LINC02726
Hypermethylated CpGs					
20	48625774	7.43E−58	2.81E−53	18.09	LOC105372646
2	9306946	1.95E−51	5.27E−47	20.47	ASAP2
1	147078143	1.05E−50	2.49E−46	27.79	LOC105371231
15	93041590	9.83E−50	1.86E−45	4.39	RGMA
1	24207420	5.60E−49	8.83E−45	19.93	LINC02800
7	98297732	3.63E−47	4.29E−43	20.61	BRI3
13	98064461	7.26E−45	8.07E−41	19.89	LOC107984566
19	2803229	2.72E−43	2.71E−39	19.42	THOP1
20	30283700	2.82E−40	2.32E−36	14.77	LINC01597
9	92254925	3.84E−36	2.50E−32	15.40	MIR3651
20	48625774	7.43E−58	2.81E−53	18.09	LOC105372646

Chr, chromosome

The DMCs were evenly distributed across all autosomes. As depicted in Fig. 1b, the majority of the DMCs were in promoter (49.07%) or intronic (23.74%) regions. Also, most of the identified DMCs were in CpG island (45.15%) or CpG shores (21.98%). The DMCs were annotated to or near to 12,063 genes, and these genes were used for significant enrichment.

### Gene ontology enrichment analysis based on CPM phenotype in PFCs

**Fig. 4:**
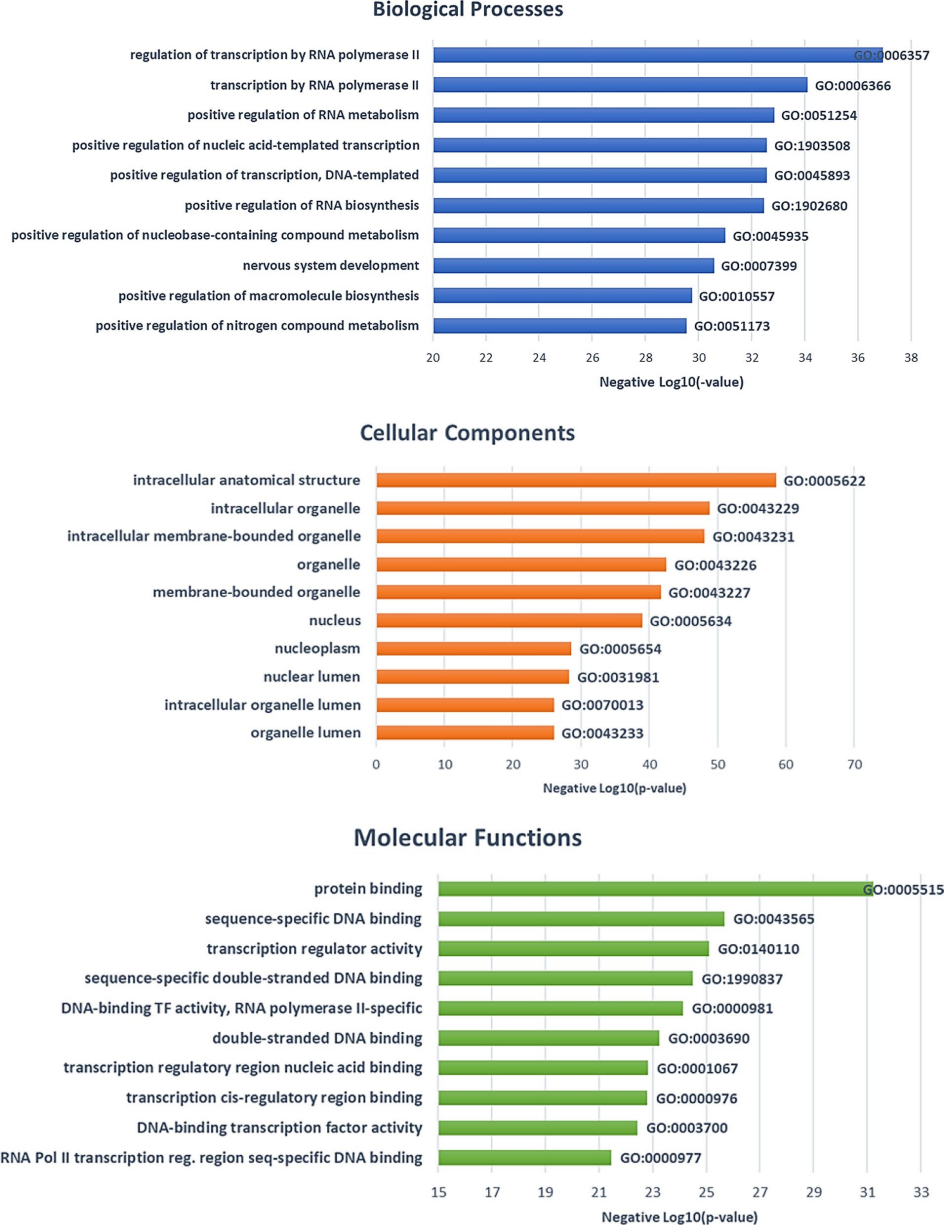
Top 10 GO term enrichment results of DMCs annotated genes between efficient and deficient conditioned pain modulation in pain free controls. Notes: All depicted GO terms were statistically significant (*p* < 0.05)

After correcting for multiple testing, DMCs annotated genes enriched 513 GO terms: 387 biological processes, 69 cellular components, and 57 molecular functions (*p* < 0.05). The top 10 GO terms categorized into biological processes, molecular functions, and cellular components are depicted in Fig. 4. Some of the key GO terms with high enrichment scores included intracellular anatomical structures (GO: 0005622; adj *p* = 2.74 × 10^–59^), regulation of transcription by RNA polymerase II (GO: 0006357; *p* = 1.13 × 10^–37)^, nervous system development (GO: 0007399; adj *p* = 2.60 × 10^–31^), and protein binding (GO: 0005515; adj. *p* = 5.80 × 10^–32^). GO terms over-represented by hypo- and hyper-methylated CpGs annotated genes are provided in Additional file 1: Figures S5 and S7.

### Functional pathway enrichment analysis based on CPM phenotype in PFCs

**Fig. 5:**
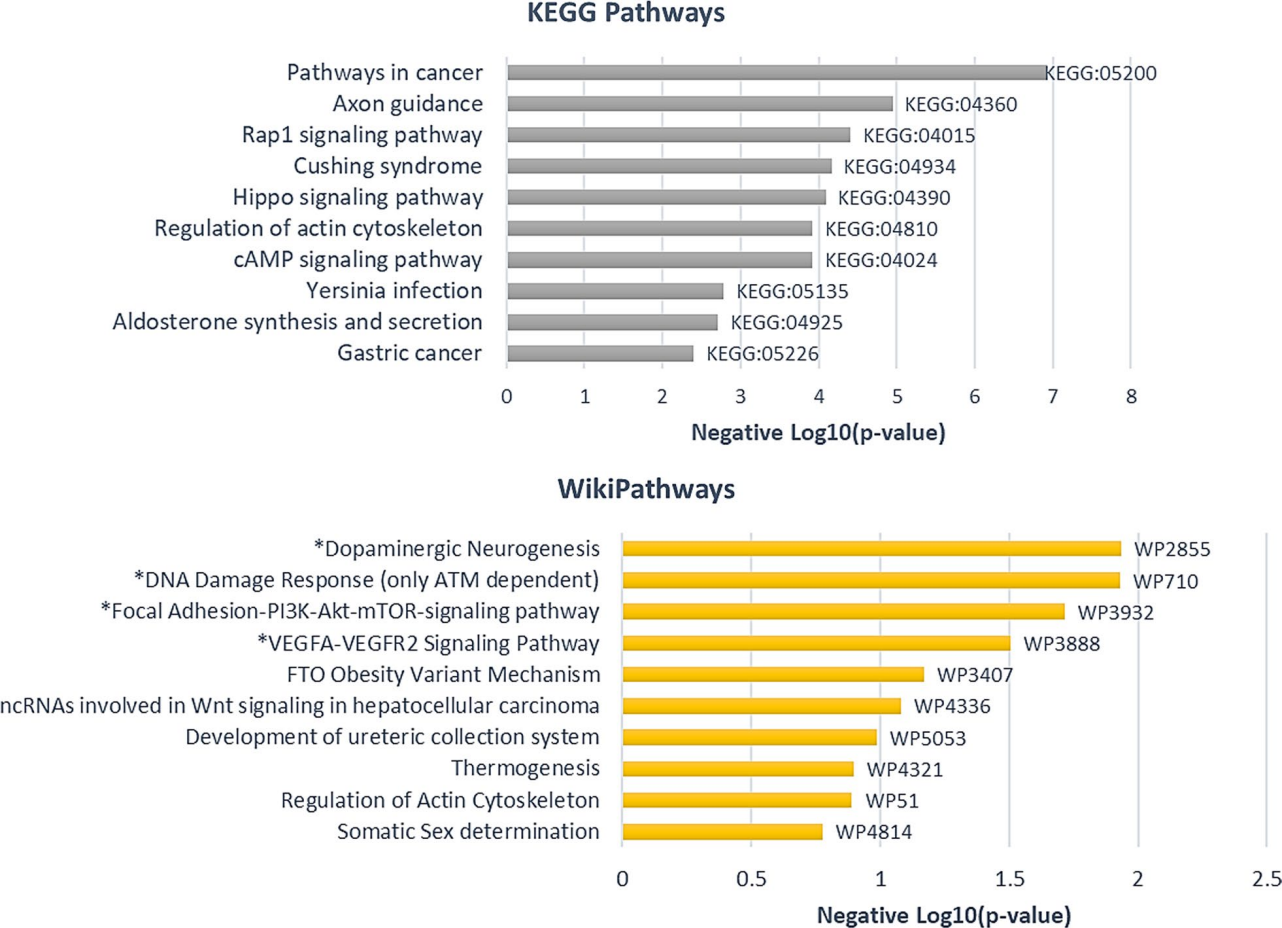
Top 10 KEGG and WikiPathways enrichment results of DMCs annotated genes between efficient and deficient conditioned pain modulation in pain free controls. Note: All depicted KEGG pathways were statistically significant and * depicts statistically significant Wikipathways (*p* < 0.05)

In adults without cLBP, functional pathway analysis revealed 28 pathways (25 from KEGG and 4 from wikipathways) enriched by DMCs annotated genes in those with efficient CPM compared with deficient CPM. Some of the enriched pathways of relevance to pain pathology included pathways involved in axon guidance (KEGG: 04360; adj. *p* = 1.13 × 10^–5^), Rap1 signaling (KEGG: 04360; adj. *p* = 3.97 × 10^–5^), Hippo signaling (KEGG 04390; adj *p* = 8.15 × 10^–5^) and dopaminergic neurogenesis (WP2855; *p* = 0.01) (Fig. 5). Pathway enrichment analysis for gene annotated hypo- and hyper-methylated CpGs are provided in Additional file 1: Figures S6 and S8.

### Comparison of enriched GO and functional pathways in cLBP and PFC groups

**Fig. 6:**
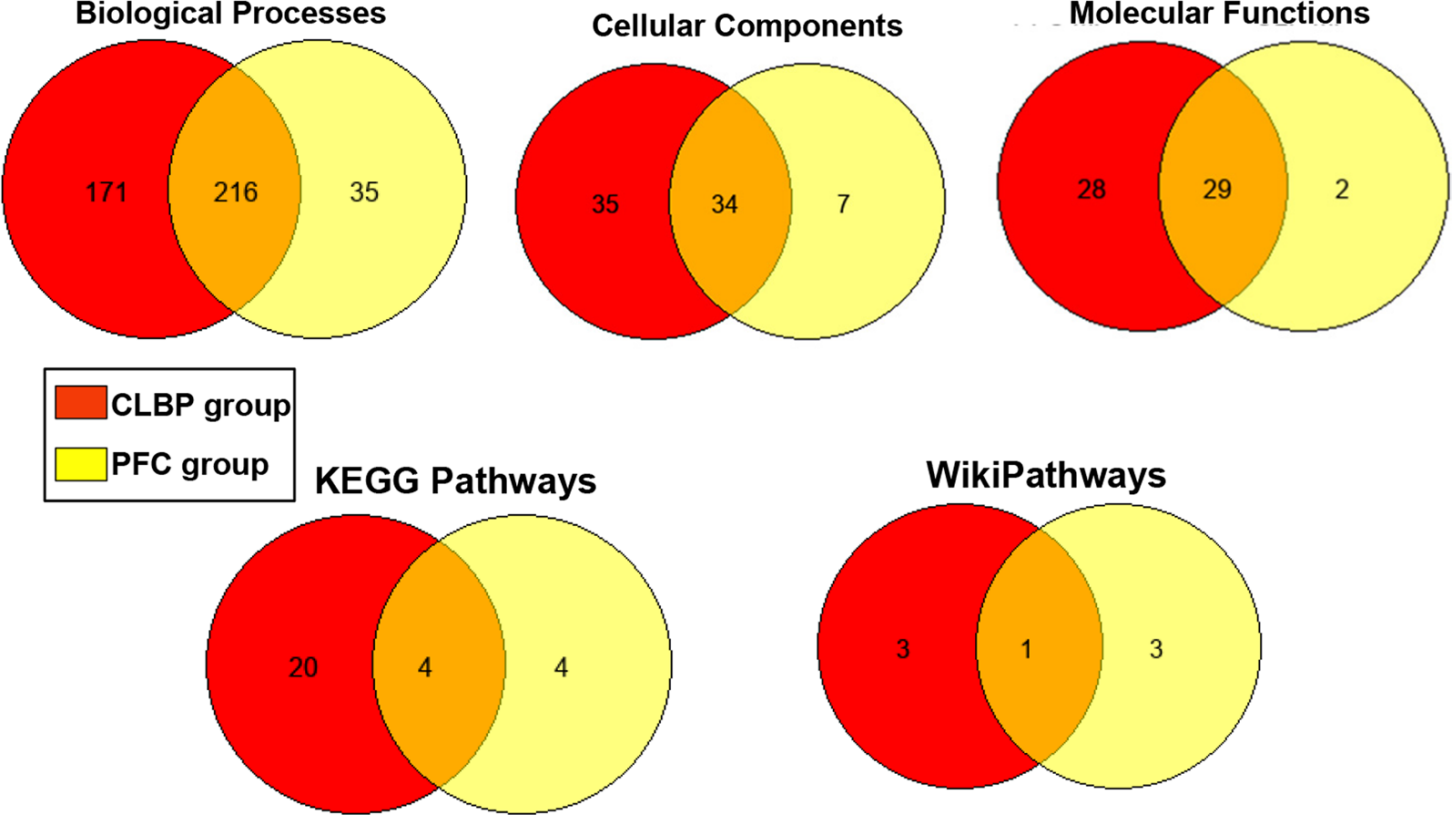
Overlap of significantly enriched (*p* < 0.05) gene ontologies (GO) and functional pathways from annotated differentially methylated genes (DMG) in chronic low back pain (CLBP) and pain-free control (PFC) groups. Venn diagram shows numbers of unique and overlapping biological processes, cellular components, molecular functions, KEGG pathways, and wikipathways enriched by annotated differentially methylated genes between efficient and deficient conditioned pain modulation comparing CLBP to PFC groups. The red circle represents the number of significant GO or pathways within the PFC group; the yellow circle represents the number of significant GO and pathways within the CLBP group

To understand the relationship between CPM efficiency and cLBP, we compared the number of GO and functional pathways enriched in the cLBP to PFC groups. As depicted in Fig. 6, more GO and functional pathways were enriched in the PFC than cLBP group, and fewer GO and functional pathways were unique to the cLBP group. In addition, there were major overlaps in GO but not functional pathways between the groups. Specifically, the fraction of overlapping GO and functional pathways were 51% biological processes, 45% cellular components, 49% molecular functions, 14% KEGG pathways, and 14% wikipathways.

## Discussion

Nonspecific cLBP is a multi-factorial, polygenic condition that affects millions of individuals worldwide. The mechanism of nonspecific cLBP has been reported to involve complex processes involving descending pain inhibitory pathways and differential DNAm of genes in pain-relevant pathways [9, 12–15, 26]. Here, we aimed to understand the difference in the DNAm landscape between individuals with nonspecific cLBP who have efficient versus deficient CPM responses. For comparison, we also examined differential methylation between efficient and deficient CPM in PFCs. Key findings of this study, keeping in mind the relatively limited size of the cohorts, suggest the existence of efficient and deficient CPM-related epigenetic signatures. The inclusion of PFCs in this study makes the interpretation of the results particularly interesting and suggests a possible role in cLBP pathology.

Among individuals with cLBP, we identified 5336 DMCs (the majority being hypomethylated) that annotated to essential genes, over-represented in functional pathways of relevance to chronic pain. Some differentially methylated genes such as *OPRM1* [42], *ADRB2* [43], *CACNA2D3* [44], *GNA12* [45], *LPL* [46],* NAXD* [46, 47], and *ASPHD1* [47] have previously been linked with various pain conditions. Generally, the involvement of a differentially methylated gene in a functional enrichment pathway validates the relevance of that association with either efficient or deficient CPM. Given the high number of annotated genes, we focus this discussion on biological processes and functional enrichment pathways of relevance to pain.

Few pathways of relevance to pain were significantly enriched in the cLBP but not the PFC group, including *MAPK Signaling* and *Ras Signaling* Pathways. The genes differentially methylated by CPM efficiency among individuals with cLBP included *DNMT3a*, *CREB1*, and *DMRT3*, which are involved in DNA methylation and transcription [48–50]. MAPK and Ras signaling pathways play an important role in intracellular signal transduction, regulating neuronal plasticity, inflammatory response, and pain sensitivity [51, 52]. Animal studies have shown that injuries (stress and nerve injury) and pain mediators activate MAPK and Ras signaling, which upregulates the release of substance P and inflammatory cytokines via NK-1 receptor activation [52]. In addition, MAPK and Ras signaling pathways are activated by neuronal TNF receptors, which induce neuronal sensitization by enhancing the release of inflammatory cytokine. Accumulating evidence also suggests that DNA methylation of genes in MAPK signaling pathways alter inflammatory cytokine levels [57] and inhibition of MAPK decreases the expression of GABA receptors [53]. Moreover, MAPK-Ras signaling pathways have been shown to play a role in signal transduction following the activation of opioid receptors [54]. As a result, targeting this pathway has been suggested to treat painful conditions, including degenerative disc disease [52, 55]. Thus, we suggest that among individuals with cLBP, CPM efficiency may be related to MAPK and Ras signaling pathways through differential methylation. However, the role of MAPK and Ras signaling pathways in CPM efficiency related to cLBP pathogenesis is still unknown, and further research is needed.

CPM efficiency includes inhibitory and facilitatory neuronal mechanisms involving the periaqueductal gray, rostral ventromedial medulla, locus coeruleus, and subnucleus reticularis dorsalis [56]. According to our data, differentially methylated genes (based on CPM efficiency) significantly enriched biological processes involved in the generation of neurons, nervous system development, neuron differentiation, and neurogenesis, among others. This finding is – at least in part- in line with the notion that epigenetic modification of genes involved in neuronal development and differentiation may play a role in central pain processing. Interestingly, these biological processes were enriched in both cLBP and PFC individuals.

Similarly, the *Hippo Signaling pathway* was significantly enriched in cLBP and PFC groups. This pathway plays a crucial role in neurodegenerative diseases and neuronal function by regulating neuronal growth and proliferation, differentiation, synaptogenesis, and apoptosis [57, 58]. In animal models, Li and colleagues found that the expression of Hippo signaling pathway components (Yap and TAZ) play an essential role in neuropathic pain by regulating synaptic and structural plasticity [59]. Hippo signaling (through YAP) also mediates the progression of osteoarthritis by inflammatory cytokines [60]. Stress stimuli and cellular stress responses are two epigenetic modification-dependent mechanisms that activate Hippo signaling pathways and neuronal cell death [57, 61]. In the midbrain, Hippo-YAP expression appears to protect dopaminergic neurons from degeneration [62]. In addition, pathways involved in axon guidance and cholinergic synapses were significantly enriched in PFCs. Together these findings suggest that epigenetically induced alterations in neuronal development/differentiation may play a role in CPM efficiency. However, the relationship between CPM efficiency and differential methylation of genes in these neurodevelopmental pathways may not be related to the pathology of cLBP.

Dopamine is an excitatory neurotransmitter in the central nervous system that plays an essential role in many brain functions that control perceptive and emotional aspects of chronic pain, including mood, motivation, voluntary movement, sleep, and perception of punishment and reward. Our findings suggest that annotated differentially methylated genes in adults with efficient versus deficient CPM enriched the dopaminergic neurogenesis pathway. Functional magnetic resonance imaging studies suggest that increase connectivity in the mesocortical and mesolimbic (dopaminergic) systems correlate with higher cLBP severity [63]. Also, dysfunctional connectivity in the mesolimbic dopaminergic system mediates the association between mechanical pain sensitivity and cLBP severity [63]. Likewise, activation of the dopaminergic pathway causes reward and analgesia, while inactivation results in depressive symptoms and hyperalgesia [64, 65]. As a result, many chronic pain conditions, including cLBP, are managed with medications that affect the dopaminergic pathway [64]. Collectively, our results and existing literature suggest that epigenetic modifications of genes in dopaminergic signaling pathways may play a role in CPM efficiency [66]. However, the enrichment of dopaminergic neurogenesis by differentially methylated genes in CPM efficiency does not appear to be unique to cLBP.

In the present study, we separated individuals with cLBP from PFCs for analysis because of clinical implications. However, we did not identify any significant differences in the proportion of efficient versus deficient CPM within the cLBP and PFCs groups. Also, we did not observe any significant differences in subjective pain measures (BPI pain severity and interference) between efficient and deficient CPM in individuals with cLBP. Our findings are consistent with recent systematic reviews that revealed a lack of strong relationship between CPM efficiency and cLBP [17, 67]. Three additional observations from our data may support this observation: (1) more pathways were enriched in the PFC than cLBP group, (2) there was more overlap in GO and functional pathways between PFC and cLBP groups than unique cLBP pathways, and (3) the PFC group had more unique pathways than cLBP group. These findings bring to light the complex relationship between CPM efficiency and clinical pain. Some investigators have suggested that CPM efficiency predicts cLBP, while others believe cLBP may be decreasing the pain modulatory mechanism. Still, others have postulated that while the “experience of pain may change the balance between pronociceptive and antinociceptive endogenous pain mechanisms,” CPM efficiency may be independent of the clinical manifestation of pain [67]. As previously mentioned, our data suggest that CPM efficiency may be related to epigenetic changes, but these changes may not be unique to cLBP. While additional studies are essential, these observations underscore the importance of including PFCs in translational studies.

### Strengths and limitations

Our study has several strengths and limitations. First, to our knowledge, this is the first evidence of the correlation between DNAm changes in alterations in CPM in adults with cLBP. The dynamic nature of DNAm changes suggests that future studies can reverse these modifications with therapeutic benefits. Also, using RRBS reduced bias otherwise associated with a candidate gene approach. Finally, the use of several advanced analyses techniques is a significant strength.

The relatively small sample size limits our study despite these strengths. Secondly, our study did not include additional validation of the differentially methylated genes. However, it provides a solid proof of concept that can be explored in larger cohort studies. Finally, the use of blood samples rather than nervous tissues is a limitation. Epigenetic changes are tissue-specific, but DNAm changes in the nervous system have been shown to correlate with changes in blood samples [68]. The use of blood samples provides a readily available marker that can be used clinically. An additional limitation of the use of blood is that methylation can vary by cell subtype. Shifts in methylation could represent a change in proportion of cell subtype populations. Accounting for cell type composition would be ideal, we will address this in future studies by either directly determining cell population sizes via flow cytometry or single cell RNAseq, or statistically using methods described previously [69–71].

## Conclusion

In conclusion, the exact etiology of nonspecific cLBP remains unknown. However, previous studies have suggested that dysfunctional regulation of endogenous pain modulatory system may play a role. For the first time, our research provides a potential novel epigenetic mechanism that differential methylation of genes involved in neuronal growth, differentiation, and plasticity may be involved in impaired CPM. Specifically, Ras and MAPK signaling pathways are significantly enriched by differentially methylated genes in CPM efficiency CPM, among individuals with cLBP. However, more pathways were enriched among PFC than cLBP group, suggesting that epigenetic-related CPM efficiency may not be related to the pathogenesis of cLBP. Therefore, our results provide a better understanding of the role of impaired CPM and central sensitization in nonspecific cLBP and suggest novel potential therapeutic targets for cLBP.

## Supplementary Information

**Additional file 1: Figure S1.** Top 10 GO terms enrichment results of annotated genes with hypomethylated CpGs between efficient and less efficient conditioned pain modulation participants with cLBP. All depicted terms were statistically significant (*p* < 0.05). **Figure S2.** Top 10 KEGG and WikiPathway enrichment results of annotated genes with hypomethylated CpGs between efficient and less efficient conditioned pain modulation in participants with cLBP. Note: None of depicted KEGG pathways are statistically significant. *WikiPathways are statistically significant. **Figure S3.** Top 10 GO terms enrichment results of annotated genes with hypermethylated CpGs between efficient and less efficient conditioned pain modulation participants with cLBP. All depicted BP, CC and *MF terms were statistically significant (*p* < 0.05). **Figure S4.** Top 10 KEGG and WikiPathway enrichment results of annotated genes with hypermethylated CpGs between efficient and less efficient conditioned pain modulation in participants with cLBP. Note:* denotes statistically significant pathways (*p* < 0.05). **Figure S5.** Top 10 GO terms enrichment results of annotated genes with hypomethylated CpGs between efficient and less efficient conditioned pain modulation in pain free control participants. All depicted terms were statistically significant (*p* < 0.05). **Figure S6.** Top 10 KEGG and WikiPathway enrichment results of annotated genes with hypomethylated CpGs between efficient and less efficient conditioned pain modulation in pain free control participants. Note: * depicts statistically significant pathways (*p* < 0.05). **Figure S7.** Top 10 GO terms enrichment results of annotated genes with hypermethylated CpGs between efficient and less efficient conditioned pain modulation in pain free control participants. All depicted terms were statistically significant (*p* < 0.05). **Figure S8.** Top 10 KEGG and WikiPathway enrichment results of annotated genes with hypermethylated CpGs between efficient and less efficient conditioned pain modulation in pain free control participants. Note: * depicts statistically significant pathways (*p* < 0.05).

**Additional file 2: Table S1.** Top 10 KEGG pathways enriched by gene annotated with DMCs between efficient and less efficient conditioned pain modulation in participants with cLBP. **Table S2.** Top 10 Wiikipathway enriched by gene annotated with DMCs between efficient and less efficient conditioned pain modulation in participants with cLBP

## Data Availability

The datasets supporting the conclusions of this article are included within the paper and its Additional files 1 and 2. All other datasets used and analyzed during the study are available from the corresponding author on reasonable request.

## Abbreviations

CPM: Conditioned pain modulation
cLBP: Chronic low back pain
CpG: Cytosine-phosphate-Guanine
DMC: Differentially methylated cytosines
DNAm: Deoxyribonucleic acid methylation
PFC: Pain-free control
RRBS: Reduced-representation bisulfite sequencing
