# Supplementary material for: Epigenome-wide DNA methylation profiling of conditioned pain modulation in individuals with non-specific chronic low back pain
Source: Clin Epigenetics. 2022 Mar 26;14:45. doi: 10.1186/s13148-022-01265-z (PMC8962463; doi:10.1186/s13148-022-01265-z)
Supplement: Supplementary file 1 — Additional file 1: Figure S1. Top 10 GO terms enrichment results of annotated genes with hypomethylated CpGs between efficient and less efficient conditioned pain modulation participants with cLBP. All depicted terms were statistically significant (p < 0.05). Figure S2. Top 10 KEGG and WikiPathway enrichment results of annotated genes with hypomethylated CpGs between efficient and less efficient conditioned pain modulation in participants with cLBP. Note: None of depicted KEGG pathways are statistically significant. *WikiPathways are statistically significant. Figure S3. Top 10 GO terms enrichment results of annotated genes with hypermethylated CpGs between efficient and less efficient conditioned pain modulation participants with cLBP. All depicted BP, CC and *MF terms were statistically significant (p < 0.05). Figure S4. Top 10 KEGG and WikiPathway enrichment results of annotated genes with hypermethylated CpGs between efficient and less efficient conditioned pain modulation in participants with cLBP. Note:* denotes statistically significant pathways (p < 0.05). Figure S5. Top 10 GO terms enrichment results of annotated genes with hypomethylated CpGs between efficient and less efficient conditioned pain modulation in pain free control participants. All depicted terms were statistically significant (p < 0.05). Figure S6. Top 10 KEGG and WikiPathway enrichment results of annotated genes with hypomethylated CpGs between efficient and less efficient conditioned pain modulation in pain free control participants. Note: * depicts statistically significant pathways (p < 0.05). Figure S7. Top 10 GO terms enrichment results of annotated genes with hypermethylated CpGs between efficient and less efficient conditioned pain modulation in pain free control participants. All depicted terms were statistically significant (p < 0.05). Figure S8. Top 10 KEGG and WikiPathway enrichment results of annotated genes with hypermethylated CpGs between efficient and less efficient condition [file 13148_2022_1265_MOESM1_ESM.docx]

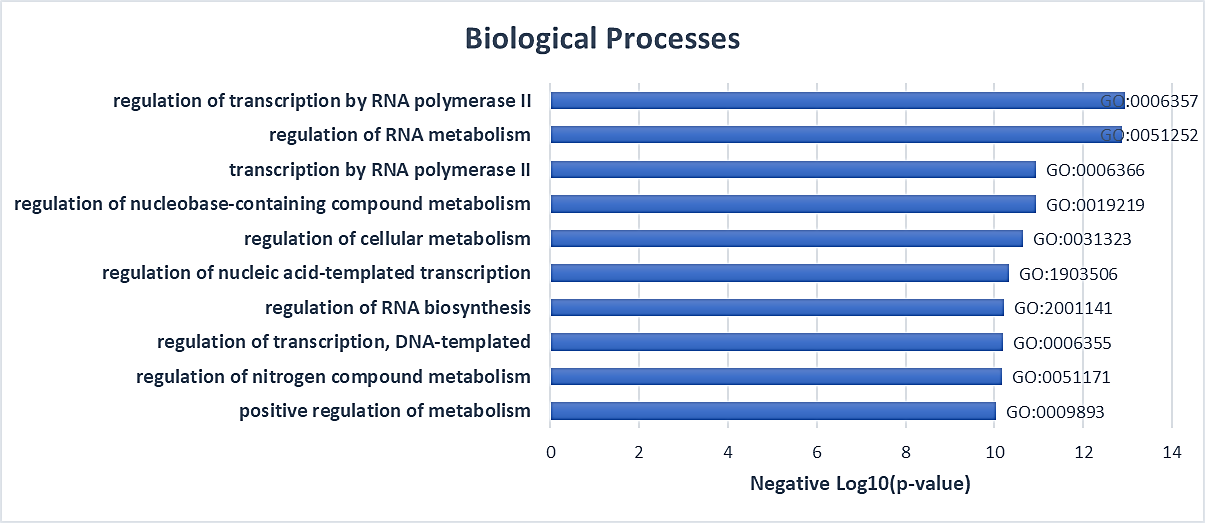


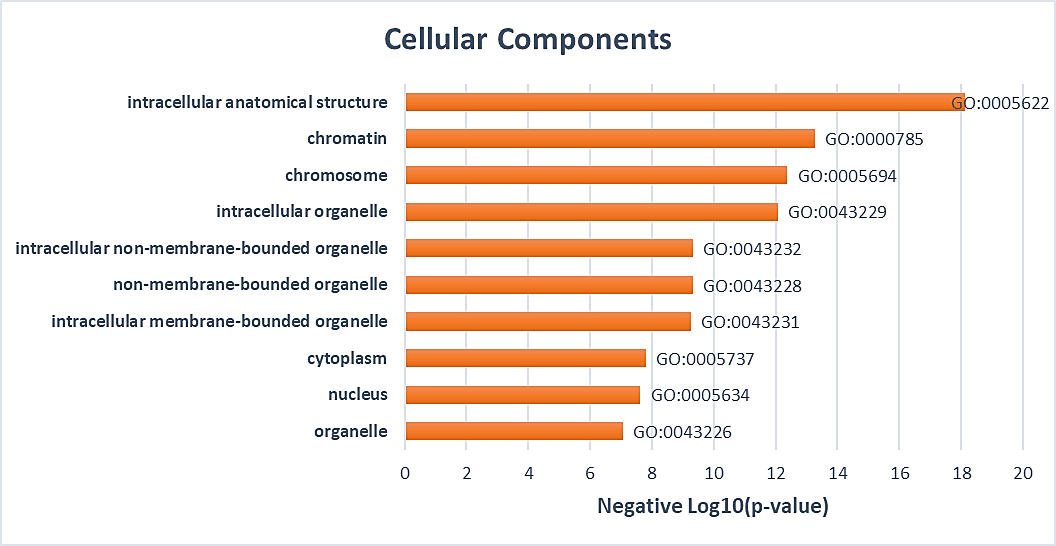


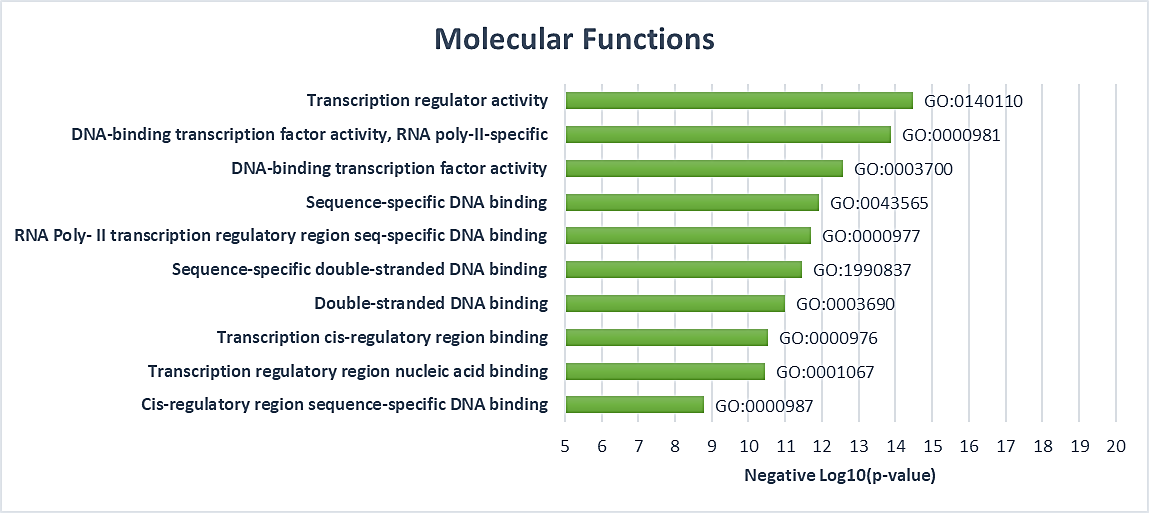


**Supplemental Figure 1.** Top 10 GO terms enrichment results of annotated genes with hypomethylated CpGs between efficient and less efficient conditioned pain modulation participants with cLBP. All depicted terms were statistically significant (p < 0.05).


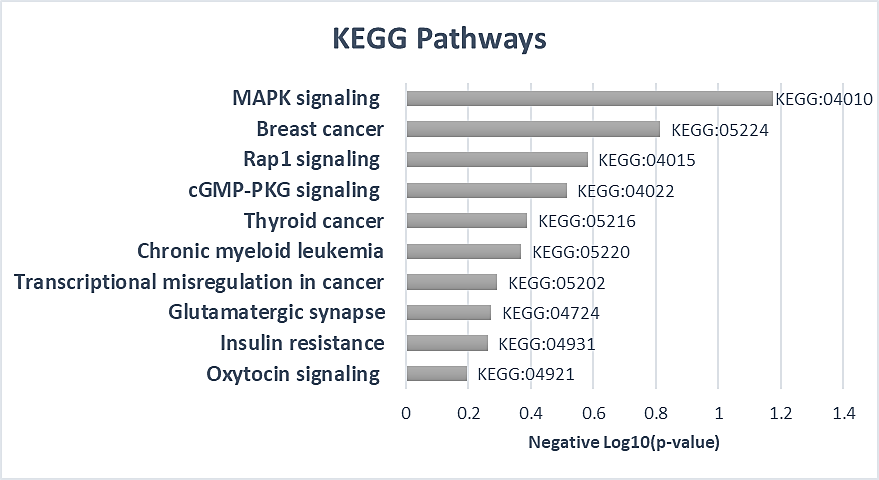


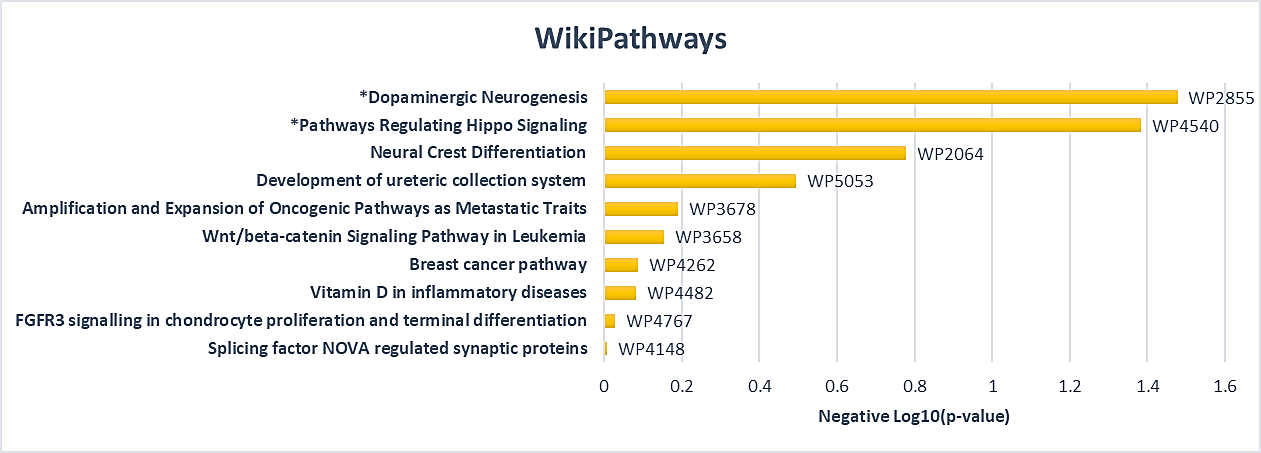


**Supplemental Figure 2.** Top 10 KEGG and WikiPathway enrichment results of annotated genes with hypomethylated CpGs between efficient and less efficient conditioned pain modulation in participants with cLBP. **Note**: None of depicted KEGG pathways are statistically significant. *WikiPathways are statistically significant


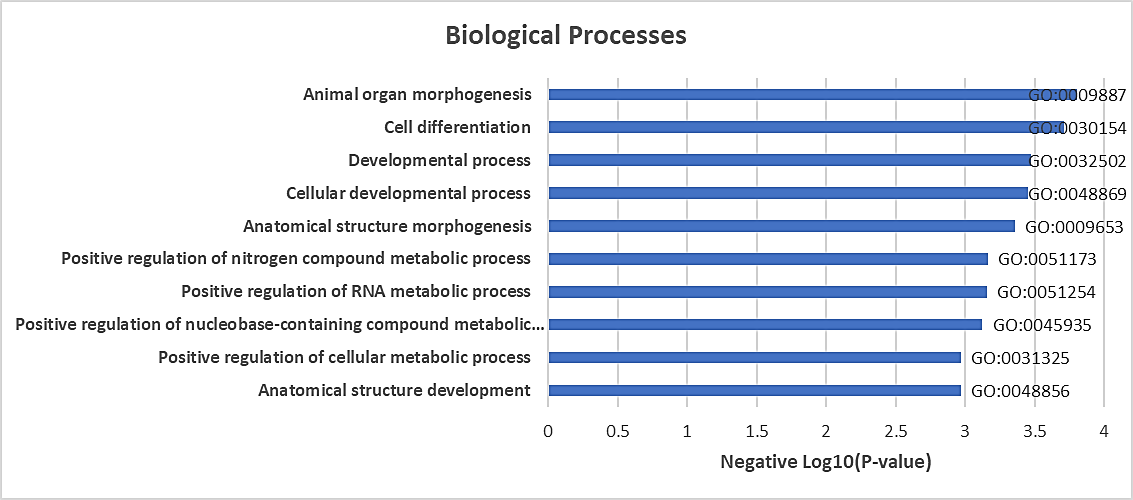


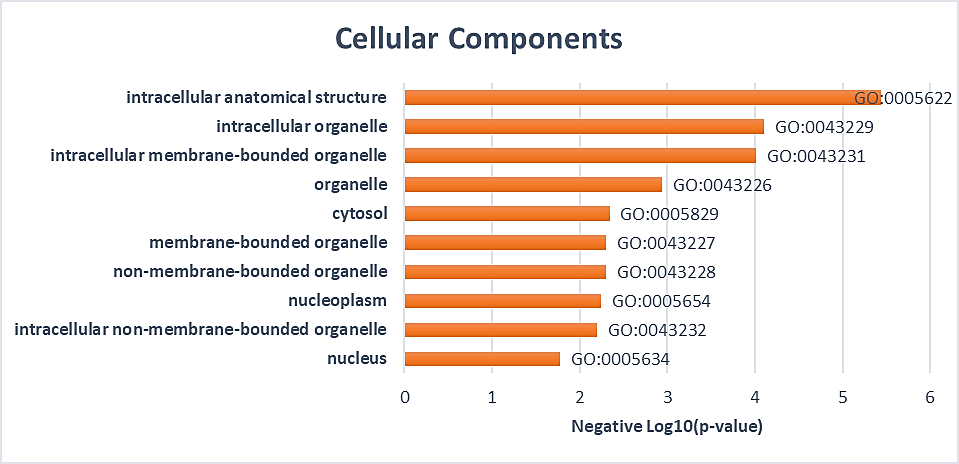


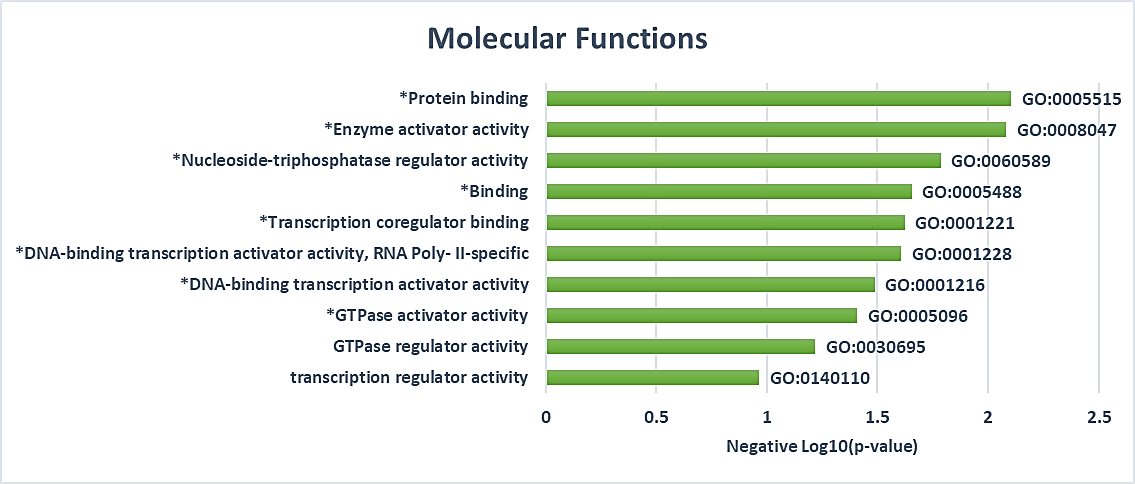


**Supplemental Figure 3.** Top 10 GO terms enrichment results of annotated genes with hypermethylated CpGs between efficient and less efficient conditioned pain modulation participants with cLBP. All depicted BP, CC and *MF terms were statistically significant (p < 0.05).


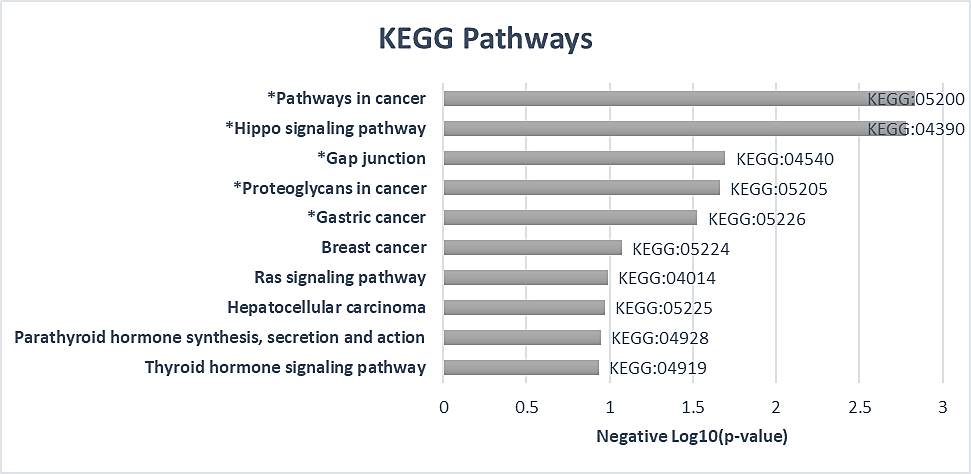


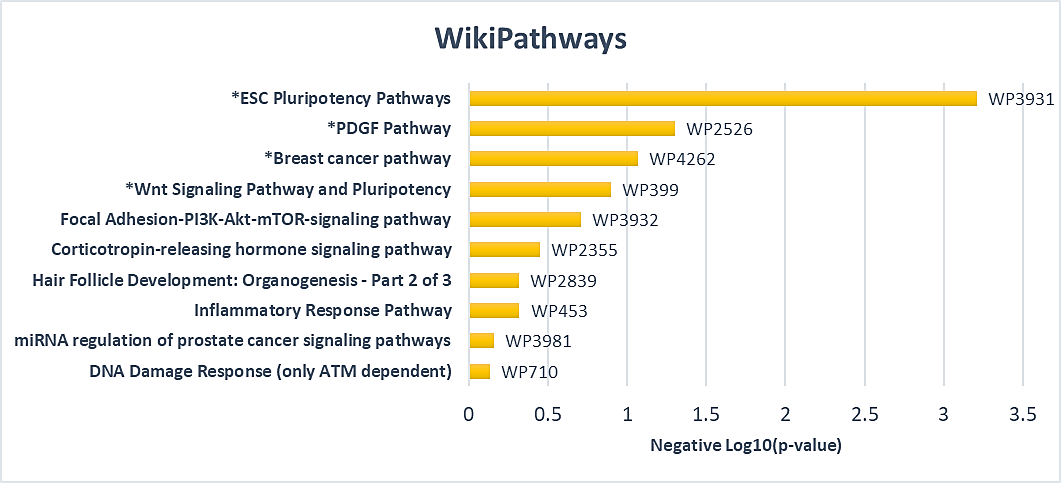


**Supplemental Figure 4.** Top 10 KEGG and WikiPathway enrichment results of annotated genes with hypermethylated CpGs between efficient and less efficient conditioned pain modulation in participants with cLBP. **Note**:* denotes statistically significant pathways (p < 0.05)


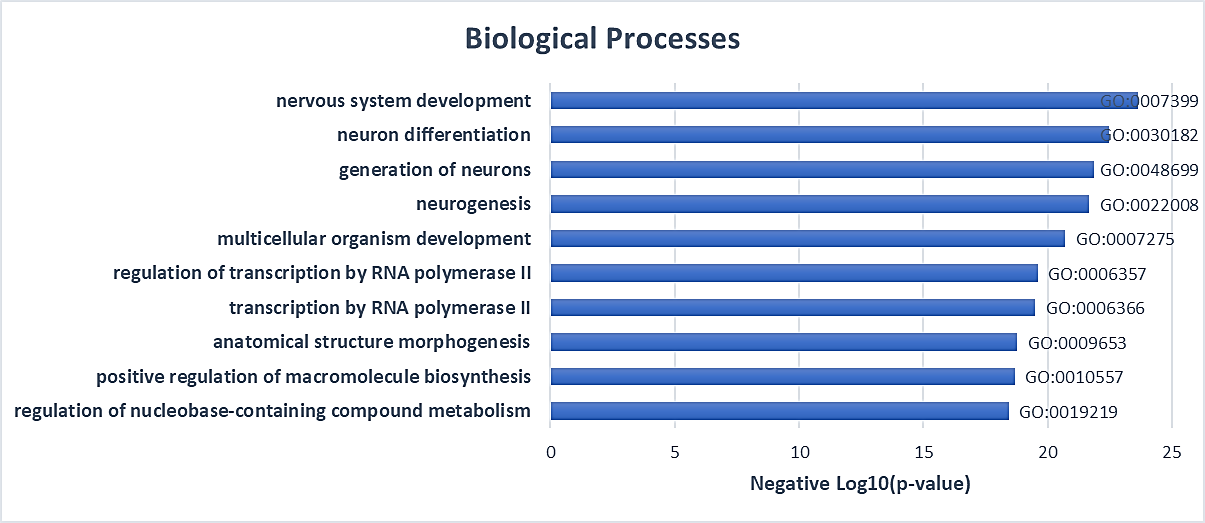


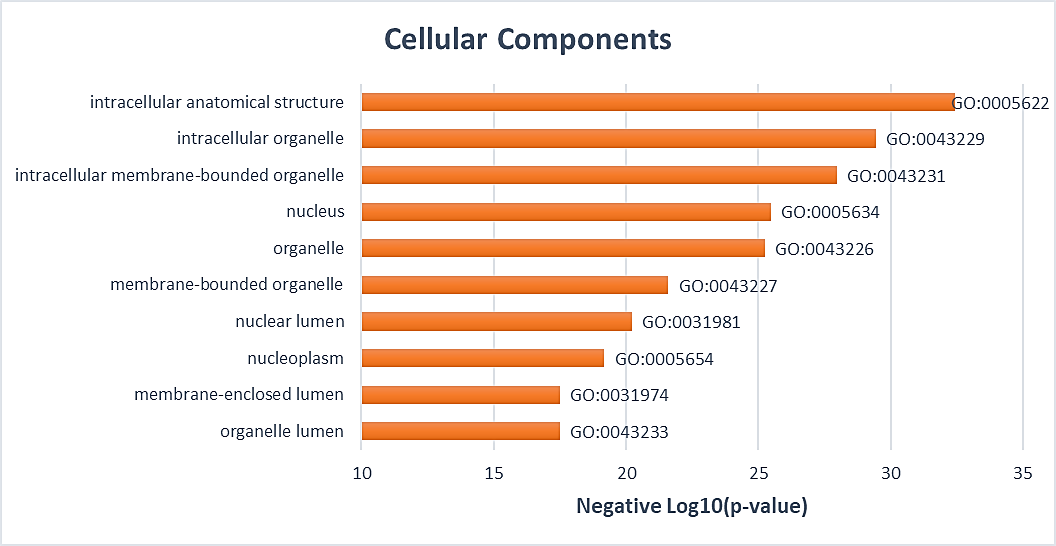


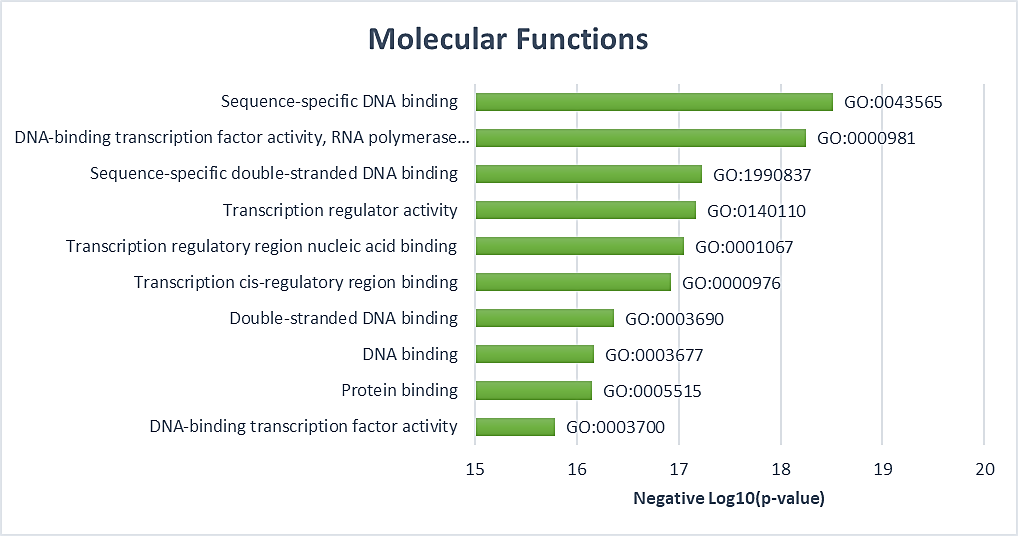


**Supplemental Figure 5.** Top 10 GO terms enrichment results of annotated genes with hypomethylated CpGs between efficient and less efficient conditioned pain modulation in pain free control participants. All depicted terms were statistically significant (p < 0.05).


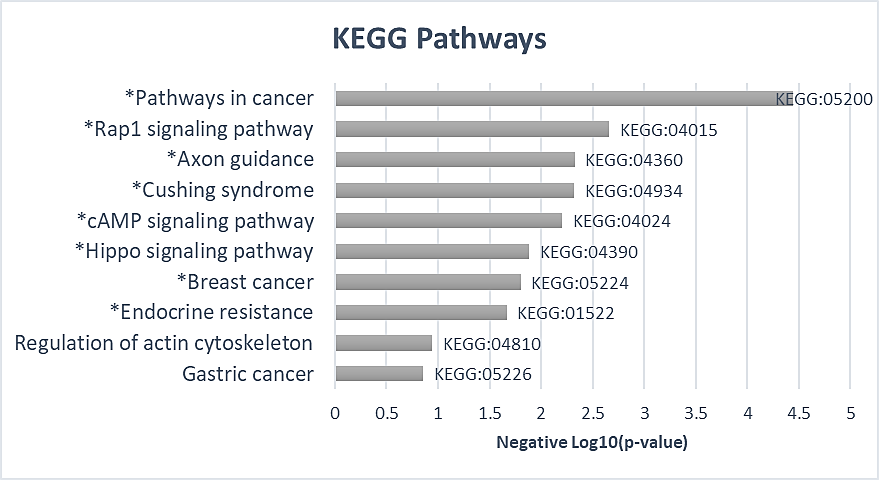


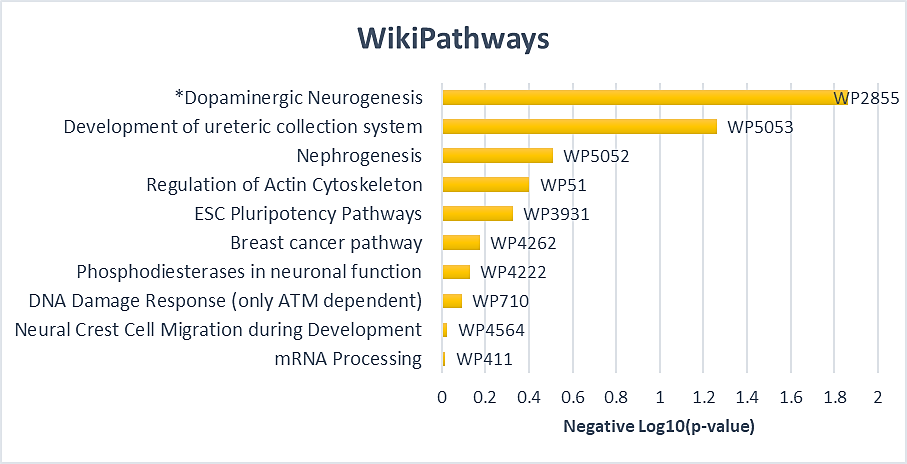


**Supplemental Figure 6.** Top 10 KEGG and WikiPathway enrichment results of annotated genes with hypomethylated CpGs between efficient and less efficient conditioned pain modulation in pain free control participants. Note: * depicts statistically significant pathways (p < 0.05).


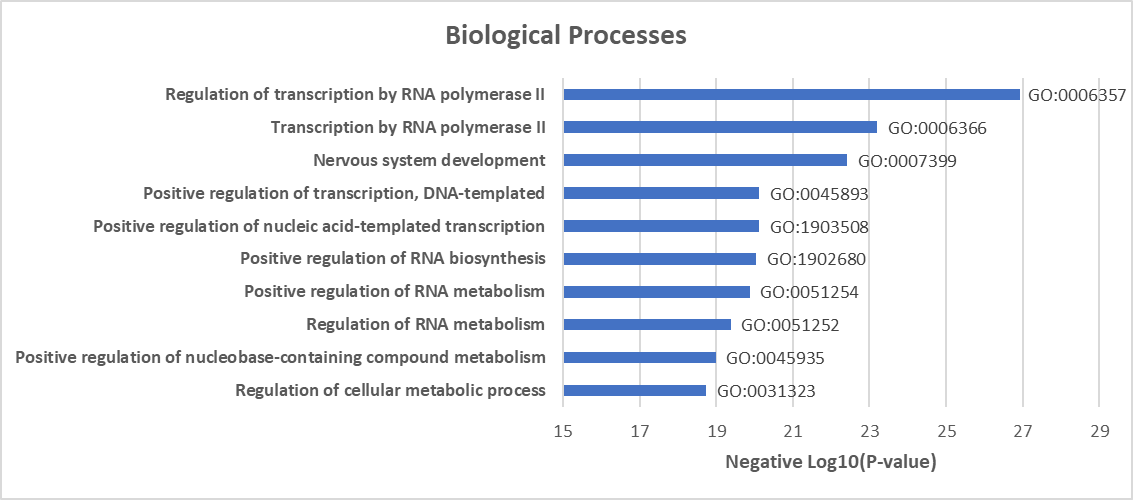


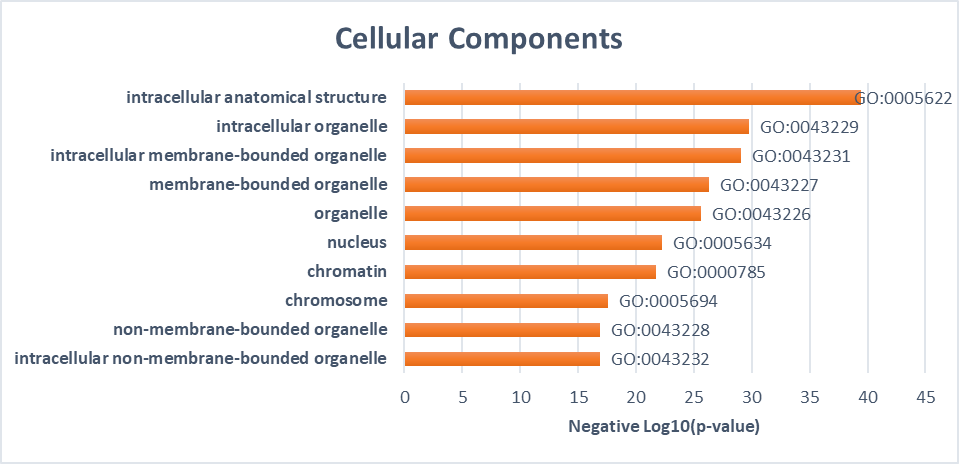


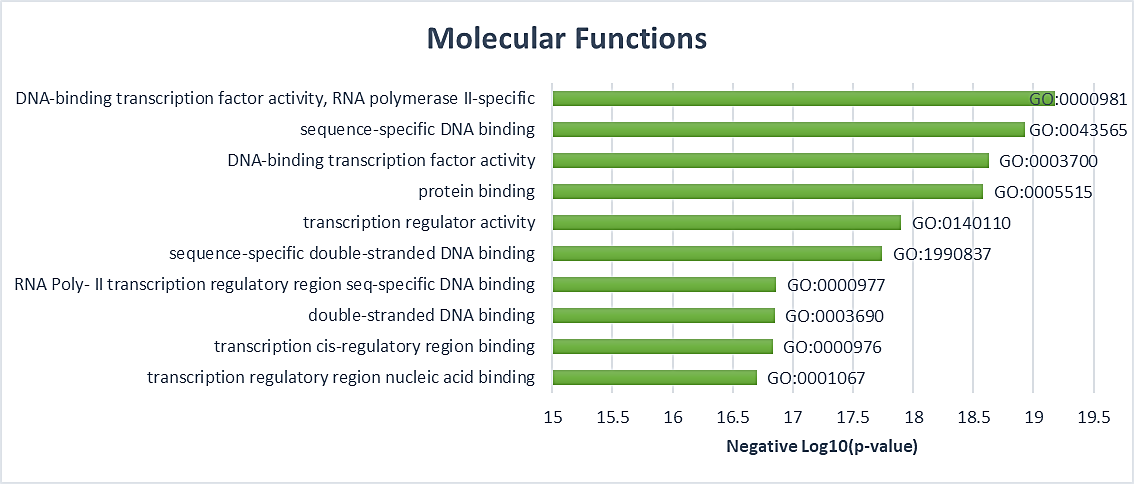


**Supplemental Figure 7.** Top 10 GO terms enrichment results of annotated genes with hypermethylated CpGs between efficient and less efficient conditioned pain modulation in pain free control participants. All depicted terms were statistically significant (p < 0.05).


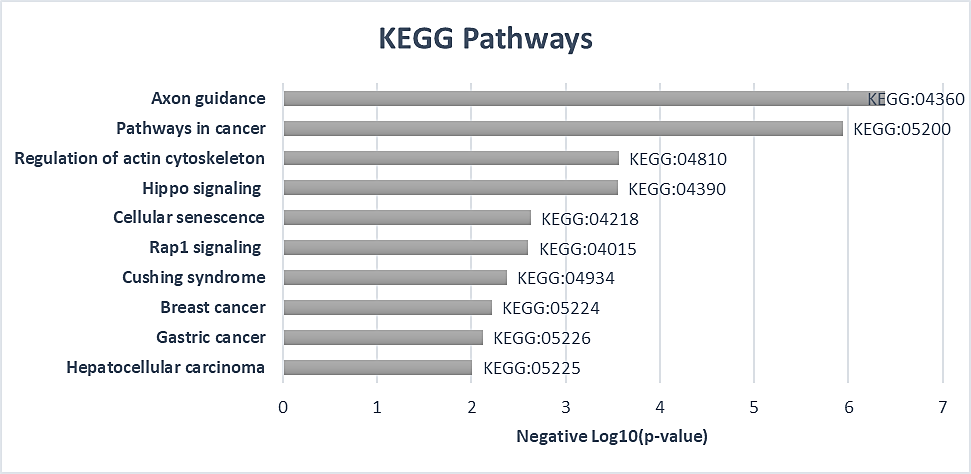


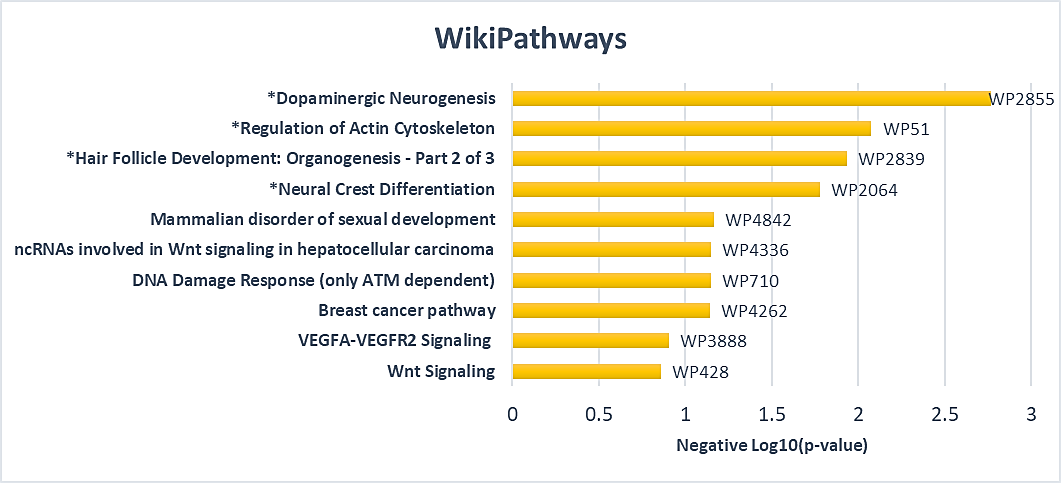


**Supplemental Figure 8.** Top 10 KEGG and WikiPathway enrichment results of annotated genes with hypermethylated CpGs between efficient and less efficient conditioned pain modulation in pain free control participants. Note: * depicts statistically significant pathways (p < 0.05).
