# Supplementary material for: Epigenome-wide DNA methylation profiling of conditioned pain modulation in individuals with non-specific chronic low back pain
Source: Clin Epigenetics. 2022 Mar 26;14:45. doi: 10.1186/s13148-022-01265-z (PMC8962463; doi:10.1186/s13148-022-01265-z)
Supplement: Supplementary file 2 — Additional file 2: Table S1. Top 10 KEGG pathways enriched by gene annotated with DMCs between efficient and less efficient conditioned pain modulation in participants with cLBP. Table S2. Top 10 Wiikipathway enriched by gene annotated with DMCs between efficient and less efficient conditioned pain modulation in participants with cLBP [file 13148_2022_1265_MOESM2_ESM.docx]

**Supplemental Table 1:** Top 10 KEGG pathways enriched by gene annotated with DMCs between efficient and less efficient conditioned pain modulation in participants with cLBP

| **Enriched Pathway** | **KEGG ID** | **Adj. p-value** | **Term size** | **Genes** |
| --- | --- | --- | --- | --- |
| MAPK signaling pathway | 04010 | 2.22E-04 | 294 | AREG,ARRB1,EFNA2,FGF3,FGF4,GRB2,MAP2K1,MAP2K3,MAPK8IP1,MECOM,NFATC3,NGFR,PDGFA,PDGFB,PDGFRB,PPM1B,PRKACA,PRKCB,RASA1,RASGRF2,RRAS2,TGFB1,TGFBR1,CACNA1I,CACNA2D2,CACNA2D3,CACNG2,CACNG3,CRK,CRKL,CSF1,CSF1R,DUSP4,DUSP5,EFNA5,EREG,FGF1,FGF19,FGF20,FGF8,FGFR2,FLNC,FLT1,FLT4,FOS,GADD45B,GADD45G,GNA12,IGF1R,IKBKB,INSR,KDR,MAP2K2,MAP3K11,MAP3K4,MAP3K5,MAP3K8,MAPK14,MAPKAPK2,MAX,MKNK2,MYC,NFATC1,NR4A1,NTRK1,PLA2G4A,PLA2G4C,PPM1A,PPP3CA,RAF1,RASGRF1,RASGRP1,RASGRP2,RPS6KA2,SRF |
| Pathways in cancer | 05200 | 5.06E-04 | 529 | ADCY7,AXIN1,BBC3,BIRC2,CCDC6,CTBP2,CTNNB1,CUL2,E2F3,ESR1,FGF3,FGF4,FZD1,FZD5,FZD7,GNB1,GRB2,GSTO2,HEYL,IL4R,LAMA5,LAMB1,LAMC1,LRP6,MAP2K1,MDM2,MECOM,NFKBIA,PDGFA,PDGFB,PDGFRB,PLD1,PRKACA,PRKCB,RAD51,SLC2A1,TCF7,TERT,TGFB1,TGFBR1,TRAF3,WNT7B,ABL1,ADCY3,ADCY8,AGT,BCL2L11,BCR,BMP4,CALML4,CALML6,CCND1,CDH1,CDK6,CDKN1A,CRK,CRKL,CSF1R,CTBP1,CTNNA1,DLL4,EDNRB,EGLN3,EML4,EPAS1,FGF1,FGF19,FGF20,FGF8,FGFR2,FLT4,FOS,FRAT1,FRAT2,FZD10,GADD45B,GADD45G,GLI2,GNA11,GNA12,GNAI2,GNB4,GNG4,GNG7,GSTM4,GSTP1,HES1,IFNGR2,IGF1R,IKBKB,IL12RB1,JAG1,JAK3,JUP,LAMC3,MAP2K2,MAX,MGST2,MSH6,MYC,NOTCH1,NTRK1,PIM1,PLEKHG5,PTGER1,RAF1,RALBP1,RARB,RASGRP1,RASGRP2,RUNX1,RUNX1T1,RXRA,SMAD3,SMO,SPI1,TCF7L2,ZBTB16 |
| Ras signaling pathway | 04014 | 2.71E-03 | 231 | EFNA2,FGF3,FGF4,GNB1,GRB2,HTR7,MAP2K1,NGFR,PAK6,PDGFA,PDGFB,PDGFRB,PLA2G1B,PLD1,PRKACA,PRKCB,RASA1,RASGRF2,RRAS2,ZAP70,ABL1,AFDN,CALML4,CALML6,CSF1,CSF1R,EFNA5,FGF1,FGF19,FGF20,FGF8,FGFR2,FLT1,FLT4,GNB4,GNG4,GNG7,GRIN1,GRIN2B,IGF1R,IKBKB,INSR,KDR,LAT,MAP2K2,NTRK1,PAK4,PLA2G4A,PLA2G4C,PLA2G6,RAF1,RALBP1,RASA3,RASAL1,RASGRF1,RASGRP1,RASGRP2,SHC2,TIAM1 |
| Chronic myeloid leukemia | 05220 | 3.42E-03 | 76 | CTBP2,E2F3,GRB2,MAP2K1,MDM2,MECOM,NFKBIA,TGFB1,TGFBR1,ABL1,BCR,CCND1,CDK6,CDKN1A,CRK,CRKL,CTBP1,GADD45B,GADD45G,IKBKB,MAP2K2,MYC,RAF1,RUNX1,SHC2,SMAD3 |
| Breast cancer | 05224 | 5.81E-03 | 147 | AXIN1,CTNNB1,E2F3,ESR1,FGF3,FGF4,FZD1,FZD5,FZD7,GRB2,HEYL,LRP6,MAP2K1,TCF7,WNT7B,CCND1,CDK6,CDKN1A,CSNK1A1,DLL4,FGF1,FGF19,FGF20,FGF8,FLT4,FOS,FRAT1,FRAT2,FZD10,GADD45B,GADD45G,HES1,IGF1R,JAG1,MAP2K2,MYC,NOTCH1,PGR,RAF1,SHC2,TCF7L2 |
| Rap1 signaling pathway | 04015 | 9.88E-03 | 210 | ADCY7,CTNNB1,EFNA2,FGF3,FGF4,ITGB2,MAP2K1,MAP2K3,NGFR,PDGFA,PDGFB,PDGFRB,PRKCB,SIPA1,VAV2,ADCY3,ADCY8,AFDN,ARAP3,CALML4,CALML6,CDH1,CRK,CRKL,CSF1,CSF1R,EFNA5,EVL,FGF1,FGF19,FGF20,FGF8,FGFR2,FLT1,FLT4,GNAI2,GRIN1,GRIN2B,IGF1R,INSR,KDR,LAT,MAGI2,MAP2K2,MAPK14,PARD3,PRKCZ,RAF1,RASGRP2,SIPA1L2,SIPA1L3,TIAM1,VASP |
| Gastric cancer | 05226 | 1.53E-02 | 148 | AXIN1,CTNNB1,E2F3,FGF3,FGF4,FZD1,FZD5,FZD7,GRB2,LRP6,MAP2K1,TCF7,TERT,TGFB1,TGFBR1,WNT7B,CCND1,CDH1,CDKN1A,CSNK1A1,CTNNA1,FGF1,FGF19,FGF20,FGF8,FGFR2,FRAT1,FRAT2,FZD10,GADD45B,GADD45G,JUP,MAP2K2,MYC,RAF1,RARB,RXRA,SHC2,SMAD3,TCF7L2 |
| Parathyroid hormone synthesis, secretion and action | 04928 | 1.86E-02 | 106 | ADCY7,ARRB1,CYP24A1,LRP6,MAP2K1,NACA,PDE4A,PDE4D,PLD1,PRKACA,PRKCB,RUNX2,ADCY3,ADCY8,BGLAP,CDKN1A,CREB1,CREB3L2,CREB3L3,FOS,GNA11,GNA12,GNAI2,KL,MEF2A,MMP25,PDE4B,PTH1R,RAF1,RXRA,VDR |
| Transcriptional misregulation in cancer | 05202 | 4.61E-02 | 191 | BCL11B,BIRC2,CDK9,HOXA9,MDM2,NFKBIZ,NGFR,PDGFA,PROM1,RUNX2,SIN3A,ATM,BAIAP3,BCL6,CDKN1A,CEBPE,CSF1R,ERG,ETV5,ETV6,ETV7,EYA1,FLT1,GADD45B,GADD45G,H3-3A,H3C1,HHEX,HMGA2,IGF1R,JUP,MAF,MAX,MLLT1,MYC,NTRK1,PAX5,PAX7,PBX1,RUNX1,RUNX1T1,RXRA,SPI1,TCF3,TLX3,WT1,ZBTB16 |
| Hippo signaling pathway | 04390 | 5.84E-02 | 157 | AREG,AXIN1,BBC3,BIRC2,CTNNB1,FRMD1,FZD1,FZD5,FZD7,GDF7,ITGB2,LIMD1,LLGL2,PPP2R2B,TCF7,TGFB1,TGFBR1,TP73,WNT7B,BMP4,BMPR1A,CCND1,CDH1,CTNNA1,FGF1,FZD10,GDF6,GLI2,MOB1A,MYC,PARD3,PPP2R2C,PRKCZ,SMAD3,SMAD7,SOX2,TCF7L2,TEAD4,WTIP,YWHAZ |

**Supplemental Table 2:**

Top 10 Wiikipathway enriched by gene annotated with DMCs between efficient and less efficient conditioned pain modulation in participants with cLBP

| **Enriched Pathway** | **WP ID** | **Adj. p-value** | **Term size** | **Genes** |
| --- | --- | --- | --- | --- |
| Pathways Regulating Hippo Signaling | WP4540 | 0.01 | 99 | CDH4,CTNNB1,NGFR,PDGFRB,PRKACA,PRKAG2,PRKAR1B,PRKAR2A,PRKCB,TCF7,CDH1,CDH11,CDH16,CDH19,CDH3,CSF1R,FGFR2,FLT1,FLT4,GNA11,GNAI2,IGF1R,INSR,KDR,NTRK1,PRKAR2B,PRKCD,PRKCE,PRKCZ,SMAD3,TCF7L2,TEAD4 |
| Hair Follicle Development: Organogenesis - Part 2 of 3 | WP2839 | 0.02 | 33 | CTNNB1,FZD1,LAMA5,MIR4758,PDGFA,RUNX2,BMP4,CCND1,CDH1,FGF1,FGFR2,GLI2,MYC,SMO,SNAI1 |
| Dopaminergic Neurogenesis | WP2855 | 0.02 | 30 | GBX2,TGFB1,EN1,FGF8,GLI2,LMX1A,LMX1B,MSX1,NEUROG2,NKX2-2,NKX6-1,PITX3,SOX2,TH |
| Breast cancer pathway | WP4262 | 0.05 | 156 | AXIN1,CTNNB1,E2F3,ESR1,FGF3,FGF4,FZD1,FZD5,FZD7,GRB2,HEYL,LRP6,MAP2K1,PARP1,RAD51,TCF7,WNT7B,ATM,CCND1,CDK6,CDKN1A,CSNK1A1,DLL4,FGF1,FGF19,FGF20,FGF8,FLT4,FOS,FRAT1,FRAT2,FZD10,GADD45B,GADD45G,HES1,IGF1R,MAP2K2,MYC,NOTCH1,PGR,RAF1,SHC2,TCF7L2 |
| Ras Signaling | WP4223 | 0.08 | 184 | GNB1,GRB2,HTR7,MAP2K1,NGFR,PAK6,PDGFRB,PLA2G1B,PLD1,PRKACA,PRKCB,RASA1,RASGRF2,RRAS2,ZAP70,ABL1,AFDN,CALML4,CALML6,CSF1R,FGFR2,FLT1,FLT4,GNB4,GNG4,GNG7,GRIN1,GRIN2B,IGF1R,IKBKB,INSR,KDR,LAT,MAP2K2,NTRK1,PAK4,PLA2G4A,PLA2G4C,PLA2G6,RAF1,RALBP1,RASA3,RASAL1,RASGRF1,RASGRP1,RASGRP2,SHC2,TIAM1 |
| Focal Adhesion-PI3K-Akt-mTOR-signaling pathway | WP3932 | 0.09 | 309 | CAB39L,COL5A1,EFNA2,FGF3,FGF4,GNB1,GRB2,IL4R,IRS4,ITGA9,ITGB2,ITGB5,LAMA5,LAMB1,LAMC1,MAP2K1,MDM2,MIR4758,NGFR,PDGFA,PDGFB,PDGFRB,PIK3R4,PPP2R2B,RHEB,SLC2A1,CDKN1A,CHRM1,CREB1,CREB3L2,CREB3L3,CSF1,CSF1R,DDIT4,EFNA5,EPAS1,FGF1,FGF11,FGF12,FGF19,FGF20,FGF8,FGFR2,FLT1,FLT4,GHR,GNB4,GNG4,GNG7,HIF3A,IGF1R,IKBKB,INSR,IRS2,JAK3,KDR,LAMC3,MAP2K2,MIR3606,PFKFB2,PFKFB3,PIK3C2B,PIK3CG,PIK3R5,PPARGC1A,PPP2R2C,PPP2R3A,PPP2R5B,RAF1,SREBF1,STK11,STRADA,TBC1D1 |
| MAPK Signaling Pathway | WP382 | 0.10 | 249 | ARRB1,FGF3,FGF4,GRB2,MAP2K1,MAP2K3,MAPK8IP1,NFATC3,PDGFA,PDGFB,PDGFRB,PPM1B,PRKACA,RASA1,RASGRF2,RRAS2,TGFB1,TGFBR1,CACNA1I,CACNA2D2,CACNA2D3,CACNG2,CACNG3,CRK,CRKL,DUSP4,FGF1,FGF11,FGF12,FGF19,FGF20,FGF8,FGFR2,FLNC,FOS,GNA12,IKBKB,MAP2K2,MAP3K11,MAP3K4,MAP3K5,MAP3K8,MAPK14,MAPKAPK2,MAX,MIR935,MKNK2,MYC,NFATC1,NR4A1,NTRK1,PLA2G4A,PLA2G4C,PPM1A,PPP3CA,PRKCD,RAF1,RASGRF1,RASGRP1,RASGRP2,SRF |
| Neural Crest Differentiation | WP2064 | 0.12 | 102 | AXIN1,CTBP2,CTNNB1,DMBX1,GBX2,HAND1,OLIG2,TFAP2A,BMP4,CDH1,DCT,DLL4,FGF19,FGF8,FGFR2,HDAC4,HES1,ISL1,LHX2,MSX1,MYC,NOTCH1,OLIG3,PAX7,PRTG,RHOB,SNAI1,SOX9,TBX6,TFAP2B |
| ESC Pluripotency Pathways | WP3931 | 0.18 | 118 | ACVR1,AXIN1,CTNNB1,FGF3,FGF4,FZD1,FZD5,FZD7,GRB2,HNF1A,LRP6,MAP2K1,MAP2K3,MDM2,PDGFA,PDGFB,PDGFRB,WNT7B,ACTR2,BMP4,BMPR1A,FGF1,FGF11,FGF12,FGF19,FGF20,FGF8,FGFR2,FOS,MAP2K2,RAF1,SMAD6,SMAD7 |
| Wnt/beta-catenin Signaling Pathway in Leukemia | WP3658 | 0.28 | 26 | CTNNB1,LRP6,BCL9,CCND1,CSNK1A1,JUP,MYC,PYGO1,RUNX1T1,TCF3,ZBTB16 |
